# The gut microbiota‐astrocyte axis: Implications for type 2 diabetic cognitive dysfunction

**DOI:** 10.1111/cns.14077

**Published:** 2023-01-04

**Authors:** Zi‐Han Li, Ya‐Yi Jiang, Cai‐Yi Long, Qian Peng, Ren‐Song Yue

**Affiliations:** ^1^ Hospital of Chengdu University of Traditional Chinese Medicine Chengdu China

**Keywords:** astrocytes, brain‐gut axis, diabetes cognitive dysfunction, gut microbiota

## Abstract

**Background:**

Diabetic cognitive dysfunction (DCD) is one of the most insidious complications of type 2 diabetes mellitus, which can seriously affect the ability to self‐monitoring of blood glucose and the quality of life in the elderly. Previous pathological studies of cognitive dysfunction have focused on neuronal dysfunction, characterized by extracellular beta‐amyloid deposition and intracellular tau hyperphosphorylation. In recent years, astrocytes have been recognized as a potential therapeutic target for cognitive dysfunction and important participants in the central control of metabolism. The disorder of gut microbiota and their metabolites have been linked to a series of metabolic diseases such as diabetes mellitus. The imbalance of intestinal flora has the effect of promoting the occurrence and deterioration of several diabetes‐related complications. Gut microbes and their metabolites can drive astrocyte activation.

**Aims:**

We reviewed the pathological progress of DCD related to the “gut microbiota‐astrocyte” axis in terms of peripheral and central inflammation, intestinal and blood–brain barrier (BBB) dysfunction, systemic and brain energy metabolism disorders to deepen the pathological research progress of DCD and explore the potential therapeutic targets.

**Conclusion:**

“Gut microbiota‐astrocyte” axis, unique bidirectional crosstalk in the brain‐gut axis, mediates the intermediate pathological process of neurocognitive dysfunction secondary to metabolic disorders in diabetes mellitus.

## Graphical abstract

The “gut microbiota‐astrocyte” axis is coupled to the pathogenesis of cognitive dysfunction secondary to type 2 diabetes mellitus. Gut microbes and astrocytes are critical factors in the gut‐brain axis, leading to diabetic cognitive dysfunction through peripheral and central inflammation, gut and blood–brain barriers (BBB), and systemic and brain energy metabolism. Gut microbiota and its metabolites are upstream drivers of astrocytic activation. The reactive astrocytes' morphology and function changes result in BBB injury, neuroinflammation, and brain energy metabolic disorder.

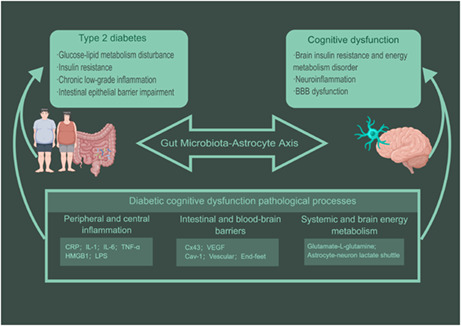

## 1 INTRODUCTION

Diabetic cognitive dysfunction (DCD) refers to the impairment of cognitive functions such as language and visual memory, information processing speed, and executive functions caused by diabetes mellitus. The 2021 American Diabetes Association (ADA) guidelines have explicitly identified diabetic cognitive impairment as a common complication of type 2 diabetes mellitus (T2DM) and indicate that the severity of cognitive impairment can deteriorate significantly over time.^1^ As population aging trends and the prevalence of diabetes continue to increase, the number of potential patients with DCD will continue to increase. The Rotterdam study found that patients with T2DM had about twice the risk of developing dementia as normal individuals, with a relative risk of 1.9, and the 95% confidence interval was 1.3–2.8.^2^ Many epidemiological studies have shown a close relationship between diabetes mellitus and cognitive impairment.^3^, ^4^ A systematic review and meta‐analysis of 144 prospective studies demonstrated that diabetes conferred a 1.25‐ to 1.91‐fold excess risk for cognitive disorders. High 2‐h postload glucose, glycosylated hemoglobin (HbA1c), and fasting plasma insulin levels were associated with an increased risk of dementia.^5^ In addition, studies suggest that mild cognitive impairment in diabetes exists in all age groups.^6^, ^7^ Cognitive development in adolescents^8^ and neurodegenerativity^9^, ^10^ in older adults show mild changes compared with controls, suggesting that the burden of cognitive dysfunction is also present in young patients.

Type 2 diabetes mellitus and Alzheimer's disease (AD) have significant overlap in risk factors and pathophysiological mechanisms. However, neuropathology studies from multiple cohort studies have suggested no association between DCD and the characteristic pathological β‐amyloid (Aβ) deposits or neurofibrillary tangle of AD.^11^, ^12^ Systemic alterations in T2DM are associated with pathophysiological mechanisms that lead to impairment of cognitive function.^13^, ^14^ Peripheral insulin resistance (IR) is thought to directly induce brain IR, and insulin signaling exerts a variety of effects in the brain regulating synaptic plasticity and peripheral energy metabolism.^15^ IR in specific brain regions was associated with decreased function of the posterior cingulate cortex and right middle temporal gyrus.^16^ Chronic hyperglycemia can induce chronic low‐grade inflammation by the accumulation of advanced glycation end products (AGEs). Nerve cell oxidative stress and inflammation are also important causes of neurodegeneration and lead to neuronal mitochondrial dysfunction. Neurons rely on the intact mitochondrial function to synthesize and secrete neurotransmitters, enhance synaptic plasticity, and maintain membrane potential. It should be noted that disorders of glucose and lipid metabolism, IR, mitochondrial dysfunction, and chronic inflammatory states are integral pathological links that together lead to endothelial damage in the cerebral microcirculation^17^, ^18^ and neurodegeneration.^19^

Prior neuroimaging studies have shown that specific functional and structural brain changes in patients with DCD are associated with cognitive impairment.^20^ T2DM accelerates the reduction of total brain volume in elderly patients.^21^ In multiple cohort studies and meta‐analyzes, differences in brain volumes related to diabetes emerge in young adulthood and increase with T2DM duration.^22^, ^23^ In the Alzheimer's Disease Neuroimaging Initiative (ADNI) cohort study, T2DM was associated with decreased global brain volume and decreased uptake of 18F‐fludeoxyglucose (18F‐FDG) in the frontal lobe, sensorimotor cortex, and striatum.^24^ In a cohort study of 713 patients with magnetic resonance imaging (MRI) and cognitive testing, T2DM‐related gray matter loss was thought to be primarily distributed in the medial temporal lobe, anterior cingulate gyrus, and medial prefrontal lobe. The white matter loss is mainly in the frontal and temporal lobes and is associated with poor visuospatial working memory (VSWM), planning capacity, and processing speed.^25^ Reduced brain volume in T2DM is associated with complex factors, including loss of neuroglia and axons, thinning and atrophy of white matter, arteriosclerosis, and venous collagen degeneration.^20^

Diffusion tensor imaging (DTI) studies have found the presence of microstructural lesions linked to white matter tissue and neural functional networks in T2DM.^26^ Decreased information processing speed in T2DM patients is associated with decreased measures of overall brain connection.^27^, ^28^ In addition, studies have shown that resting‐state functional connectivity is reduced in the default mode network and is strongly associated with HOMA‐IR.^29^ The studies of neural function networks promote the exploration of the cooperative changes among multiple functional brain areas of T2DM and further explain the effect of DCD on higher mental function.

In recent years, expert consensus and professional guidelines have called for the strengthening of early screening for DCD^30^, ^31^ and the promotion of common standardized management of cognitive impairment and glycemic control in patients with the disease,^32^ to avoid mild cognitive impairment to the deterioration of dementia. However, there is currently no evidence to support that intensive glucose control and specific Anti‐diabetic medication can prevent the progression of cognitive impairment.^33^ Emergent research directions in the gut‐brain axis as a pathological and therapeutic target of cognitive dysfunctions.^34^ Gut microbiota and astrocyte act as sensitive metabolic sensors in gut‐brain interactions. The gut microbiota is also an important upstream factor in the activation of astrocytes, which in turn promotes neuroinflammation and neurodegenerative.^35^ Large‐scale proteomic studies have shown that astrocyte activation and high expression of glycolytic proteins in the brain tissue of patients with dementia may serve as an important pathological marker of early decline in brain energy metabolism and neuroinflammation.^36^ In this review, we focus on gut microbiota and astrocyte along the gut‐brain axis and attempts to elucidate the specific course of complications associated with impaired neurocognitive function secondary to peripheral metabolic disorders in T2DM.

## 2 ASTROCYTE PLASTICITY MAINTAINS METABOLISM AND HOMEOSTASIS IN THE BRAIN

Astrocytes are the most abundant neuroglia in the brain and play important roles in maintaining brain energy metabolism, modulating cerebral blood flow, and regulating neuronal circuits. The role of astrocytes in neurocognitive function has also received considerable attention in recent years.^37^ The astrocytes are connected through a gap junction‐coupled network and its endfeet ensheath blood vessels as well as neuronal synapses. About 60% of the axon‐dendritic synapses in the hippocampus are enveloped by astrocytic processes, forming a tripartite synapse. The astrocyte endfeet connects microvessels to the neurons composing the neurovascular unit and can release vasoactive molecules, thus regulating the cerebral blood flow and BBB permeability. Horng et al.^38^ demonstrated that astrocytes in response to inflammatory signals, thus inducing tight junction (TJ) formation, limiting the number of activated T cells infiltrating the CNS.

The astrocyte, which serves as the main CNS glycogen storage cell, is coupled to the oxidative phosphorylation system (OXPHOS) metabolism of neurons through aerobic glycolysis.^39^ The astrocyte mediates the expansion of regional cerebral arteries in response to synaptic energy demands, matching blood flow to neuronal activity.^40^, ^41^ Astrocytes transfer glucose from the perivascular to the synapse to support the energy needs of neurons via glucose transporter 1 (GLUT1) and the endoplasmic reticulum pathway mediated by G6Pase‐β, G6PT, and G6Pase‐β.^42^ Under conditions of high energy demands such as glucose deprivation or intense nerve activity, and with limited energy availability such as hypoglycemia, astrocyte aerobic glycolysis can rapidly deliver pyruvate and lactic acid to maintain brain energy metabolism homeostasis.^43^ The high glycolytic activity of astrocytes results in a significant increase in the flux of the pentose‐phosphate pathway (PPP) to produce NADPH and glutathione, thus resisting neuronal oxidative stress.^44^

Astrocyte to neuron's nutritional and energetic support is essential for long‐term memory formation.^45^ The astrocyte regulates brain neuroplasticity and neurogenesis by releasing glial transmitters such as brain‐derived neurotrophic factor (BDNF) and astrocyte‐derived neurotrophic factor (ADNF).^45^ Astrocytes regulate synaptic plasticity via metabolic pathways with neurons, such as the glutamine‐glutamate cycle^46^, ^47^ and the astrocyte‐neuron lactate shuttle (ANLS),^48^, ^49^ thereby affecting memory formation. To maintain neural circuit homeostasis and thus support cognitive function, astrocytes eliminate unnecessary excitatory synaptic connections.^50^ The astrocyte also works on neural network projections. Kol et al.^51^ demonstrated that astrocytes could modulate hippocampal‐cortical communication in the anterior cingulate cortex during learning, thereby promoting the formation of remote memory.

Reactive astrocytes contribute to cognitive decline and metabolic homeostasis disorders.^27^ Astrocyte proliferation and high expression of the activation marker protein glial fibrillary acidic protein (GFAP) are hallmarks of neuroinflammation that arise with the neurodegenerative state. Astrocyte activation has been observed in various animal models of diabetes (Table 1). Zhang et al.^52^ have demonstrated that a high‐fat diet (HFD) induces upregulation of astrocyte IκK/NF‐κB, which in turn impairs astrocytic processes' plasticity. The astrocyte led to changes in extracellular GABA and BDNF in the hypothalamus, thus contributing to weight gain and impaired glucose tolerance. García‐Cáceres et al.^53^ showed that hypothalamic astrocytes regulate glucose uptake rate at the BBB by modulating insulin signaling/GLUT1, which co‐controls brain glucose sensing and systemic glucose metabolism. The potential immunometabolic mechanism makes the astrocyte activation accompanied by its metabolic plasticity damage, which leads to the attenuation of brain energy metabolism and its adaptive changes. Rahma et al.^54^ showed that the hypothalamic neuroinflammatory response in T2DM is associated with the metabolic shift from glycolysis to OXPHOS. Specific inhibition of astrocyte pyruvate dehydrogenase kinase (PDK)‐2 reduces hypothalamic inflammation and lactate levels, reversing the diabetes‐induced increase in food intake.

**TABLE 1:** Astrocyte phenotypes in experimental diabetes models.

Species (sex), age/weight	Model establish	Astrocytes		Molecular mechanisms	References
		Location	Phenotype		
C57BL/6 mice (male), 4 weeks/20–25 g	STZ (MLDS for 5 days, 50 mg/kg)	Hippocampus	GFAP↑ GRP78↓ ROS ↑ HO‐1↓	Akt↓	Wong et al.^55^
C57BL/6J mice (male), 8–10 weeks/NM	STZ (MLDS for 5 days, 40 mg/kg) or (SIJ, 150 mg/kg) + HFD	Hypothalamus	GFAP↑ Glycolytic shift↑ Lactate surge↑ TNF‐α↑ Il‐1β ↑ Il‐6 ↑	PDK2 and p‐PDH protein↑	Rahman et al.^54^
C57BL6/J mice (male), 6 weeks/NM	HFHFD for 24 weeks	Hippocampus	GFAP↑ TNF‐α IL‐1β ↑ BBB integrity ↓	NM	Takechi et al.^56^
C57BL/6 N mice (male), 6 weeks/NM	HFrD	Hippocampus	GFAP↑	NM	Yu et al.^57^
db/db mice (male), NM/NM	Spontaneous diabetes	Hippocampus	GFAP↑	Synaptophysin↓ JAK2/STAT3↑	Zhang et al.^58^
db/db mice (male), 8 weeks/NM	Spontaneous diabetes	Cortical gray matter	Astrocyte activation with detachment and retraction from mural cells	NM	Hayden et al.^59^
KK‐Ay mice (male), 3 months/NM	HFD	Hippocampus	Size of astrocytes reduced; GFAP↓ GLUT1↓ EAAT2‐BDNF↓ GDNF↓ IL‐1β TNF‐α↑	NM	Shi et al.^60^
Wistar rats (male), 3 months/200–250 g	STZ (SIJ, 45 mg/kg)	Hippocampus	GFAP ↑ S100β ↑	NM	Nagayach et al.^61^
Wistar rats (male), NM/250 g	STZ (SIJ, 70 mg/kg)	Hypothalamus	GFAP↑	NM	Lechuga‐Sancho et al.^62^
WKY rats (male), 8 weeks/160–270 g	STZ (SIJ, 75 mg/kg)	Hippocampus	GFAP‐S100B↓ GLUT1↑ GLT1‐GLAST‐GluN1↓	AGE‐RAGE	Nardin et al.^63^
SD rats (male), NM/190–240 g	STZ (SIJ, 45 mg/kg)	Hippocampus	GFAP↑ S100b↓	NM	Lebed et al.^64^
SD rats (male), NM/180–200 g	STZ (SIJ, 70 mg/kg)	MCx	GFAP↑ TNF‐α↑ IL‐1β↑	NM	Lu et al.^65^
SD rats (male), NM/200–220 g	STZ (SIJ, 60 mg/kg)	vlPAG	GFAP↑	NM	Liu et al.^66^
ICR mice (male), NM/18–22 g	STZ (SIJ, 150 mg/kg)	Hippocampus	GFAP↑ IL‐1β↑ IL‐4↑ IL‐6↑ TNF‐α↑	NM	Chu et al.^67^

*Note*: Compared with the nondiabetic group, ↓ indicates reduction, ↑ indicates increase while – indicates no statistical change.

Abbreviations: AGE, advanced glycation end products; EAAT2, recombinant excitatory amino acid transporter 2; GFAP, glial fibrillary acidic protein; GLAST, glutamate/aspartate transporter; GLT1, glutamate transporter subtype 1; GluN1, anti‐NMDA Receptor 1; GLUT1, glucose transporter 1; GRP78, glucose‐regulated protein 78; HFFD, high‐fat and high‐fructose; HFrD, high‐fructose diet; IL‐1β, interleukin‐1β; IL‐4, interleukin‐4; IL‐6, interleukin‐6; JAK2, janus tyrosine kinase 2; MCx, motor cortex; MLDS, multiple low doses; NM, not mentioned; PDH, pyruvate dehydrogenase; PDK, pyruvate dehydrogenase kinase; RAGE, recombinant receptor for advanced glycation endproducts; SD, Sprague Dawley; SIJ, single intraperitoneal injection; STAT3, signal transducer and activator of transcription 3; STZ, streptozotocin; TNF, tumor necrosis factor; vlPAG, ventrolateral region of periaqueductal gray; WKY, Wistar‐Kyoto.

## 3 INTESTINAL DYSBACTERIOSIS IS A COMMON PATHOLOGICAL FEATURE OF T2DM AND COGNITIVE DYSFUNCTION

The interplay between gut microbiota and host metabolism predisposes to drive T2DM pathogenesis through gut permeability change, chronic metabolic inflammation, IR, and metabolic energy disorder. The commonly reported results are that the genera of *Bifidobacterium*, *Bacteroides*, *Faecalibacterium*, *Akkermansia*, and *Roseburia* have a potential role in preventing T2DM, whereas *Ruminococcus*, *Fusobacteria*, and *Blautia* genera are associated with T2DM pathogenesis.^68^ A microbiome research of 123 nonobese and 169 obese Danish subjects showed that individuals with low gut microbiota abundance were more likely to be obese, IR and dyslipidemia, and the inflammatory phenotype is more pronounced.^69^ A cross‐sectional analysis of prospective cohorts from the Rotterdam study and Life Lines‐DEEP suggests that higher microbial α‐diversity and more butyrate (NaB)‐producing bacteria are associated with lower T2DM incidence and lower levels of IR.^70^

Microbial balance is essential for maintaining metabolic homeostasis and protecting cognitive function.^71^ Restoring intestinal flora balance can alleviate cognitive impairment and neuropsychiatric symptoms. A cross‐sectional study of microbiome data from 597 young Cardia patients examined the association between β‐diversity of the gut microbiota and multiple cognitive test results.^72^ A recent clinical study found a decrease in the abundance of *Bifidobacterium* and *unnamed bacteria RF39* and an increase in the abundance of *Peptidococcus* and *Leucococcus* in patients with DCD. The gut microbiota regulates calcium signaling and renin–angiotensin system in relation to DCD.^73^ Given the wide variation in gut bacterial dysregulation in DCD, further studies are required to elucidate the underlying mechanisms, and restoration of gut microbiota would be a promising therapeutic avenue for DCD.

## 4 GUT MICROBIOTA AND ITS METABOLIC PRODUCTS TARGET ASTROCYTES

**FIGURE 1:**
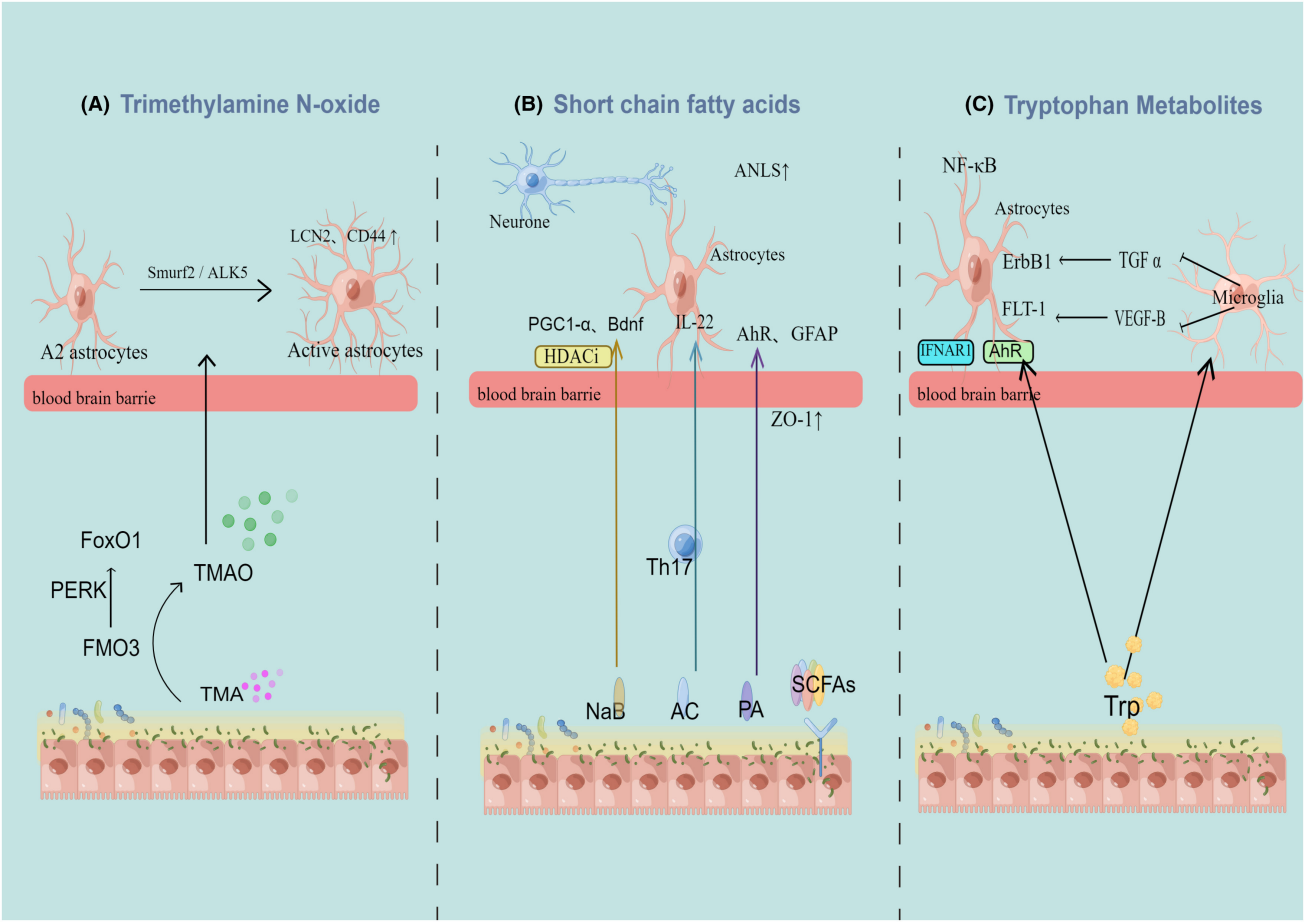
Gut microbiota metabolites drive astrocyte phenotypes. Trimethylamine N‐oxide could increase the number of reactive astrocytes and change the marker of reactive activated LCN2 and CD44 protein through the Smuef2/ALK5 axis. Improvement of short‐chain fatty acids ratio inhibited astrocytic activation and proinflammatory phenotype. The expression of PGC1‐α and brain‐derived neurotrophic factor in female mice was increased by HDACi, while the function of astrocyte mitochondria and ANLS were improved by butyrate (NaB). A significant correlation between acetate and aryl hydrocarbon receptors (AhR) and GFAP expression was observed in male mice, and the BBB structure was improved. Propionate promotes higher glycolysis and mitochondrial respiration in astrocytes and increases IL‐22 expression in male mice. AhR promotes the production of TGF‐α and VEGF‐B by microglia to indirectly regulate the transcriptional program of astrocytes. In combination with tryptophan‐derived metabolites, IFN‐I signaling activates in astrocytes and inhibits neuroinflammation.

Intestinal microflora regulates the development and function of astrocytes through neural, endocrine, and immune pathways. The gut microbiota is regulated by genetic and environmental factors, referred to as the second brain, and is also an important shaper of the intestinal microenvironment. Microbial metabolites are important information mediators of the dialogue between gut and brain, most of which can cross the BBB and act directly on the neural microenvironment, and also drive the activation of peripheral immune cells and resident neuroglia of the brain.^74^ By expressing pattern recognition receptor receptors (PRRs), such as toll‐like receptors (TLRs) and nod‐like receptors (NLRs), astrocyte constantly detects microbial‐associated molecular patterns (MAMPs) in the neural microenvironment in response to gut bacteria‐derived stimuli and initiate innate immune responses.^75^, ^76^ Astrocytes express major histocompatibility complex class II antigens and costimulatory molecules that activate T cell, thus exacerbating neuroinflammation.^77^ Short‐chain fatty acids (SCFAs), trimethylamine N‐oxide (TMAO), and aryl hydrocarbon receptors (AhR) ligands are significantly related metabolites of T2DM, and also have the potential to activate astrocytes (Figure 1).

### 4.1 Short‐chain fatty acids

Short‐chain fatty acids generated by colonic digestion and fermentation of dietary fiber have several roles within in gut microbiota‐astrocyte axis, including maintaining energy and glucose homeostasis, relieving inflammation of CNS, and regulating the secretion of neurotransmitters to ameliorate neurodegeneration. SCFAs regulate central satiety and insulin secretion through AMPK signaling, GPCR‐dependent pathway, and histone deacetylase inhibition, and influence immune cells and neuroglia to exert beneficial metabolic modulation.^78^ The regulation of short‐chain fatty acid on astrocytes is gender‐related.^79^ A decrease in acetate (AC)‐producing bacteria was found in streptozocin (STZ)‐induced mice, resulting in a decrease in hippocampal synaptophysin and learning and memory.^80^

A variety of short‐chain fatty acids cause different changes in astrocyte metabolism and immune function. According to Cuervo‐Zanatta et al.,^81^ the ratio of propionate (PA)and NaB was altered in Tg mice but recovered to control values after plant‐based diet rich in  soluble fiber feed intake, inhibiting astrocyte activation, and ameliorated neuroinflammation. PA promotes higher glycolysis and mitochondrial respiration in astrocytes further, promoting neuroinflammation, whereas NaB induces more quiescent metabolism with anti‐inflammatory actions. Sodium NaB promotes astrocyte differentiation into the neuroprotective A2 subtype, improves astrocyte mitochondrial function, and promotes the ANLS.^82^ PA treatment recuperated the astrocyte‐microglia bidirectional interplay impairment, thereby increasing the level of GFAP and restoring ZO‐1 protein increased to the level of the control group.^83^ AC serves as a specific energy substrate and metabolic marker for astrocyte.^84^ Studies using acetate 1‐c‐11 electron emission tomography have shown that activation of astrocytes is closely tied with demyelination and loss of neuron axons.^85^

### 4.2 Trimethylamine N‐oxide

Trimethylamine N‐oxide is an influential mediator of gut‐brain metabolic interaction. Multiple evidence supports TMAO as a common risk factor for cognitive function^86^, ^87^ and metabolic syndrome.^88^ The nutrients such as l‐choline, carnitine, and betaine in the high‐choline diet are decomposed into trimethylamine (TMA) by intestinal flora Trimethylamine lyase, flavin‐containing monooxygenase 3 (FMO3) is oxidized to TMAO by the liver after entering the portal vein. Meanwhile, a high‐fat diet indirectly increases circulating TMAO concentrations by causing intestinal mucosal inflammation, disrupting the hypoxic environment of the colon, increasing the abundance of gut bacteria, and promoting the catabolism of choline by the microbiota.^89^

The deterioration of insulin sensitivity and glucose homeostasis in T2DM was correlated with the increase in plasma TMAO concentration. Meta‐analysis results showed that for every 5 μmol/L increase in TMAO in plasma, the prevalence of diabetes increased by 54%.^90^ Hepatic FMO3 expression is increased in animal models of obesity and IR in human samples. IR can increase plasma TMAO concentration by promoting the FMO3 pathway.^91^ TMAO binds the liver PERK, which induces the transcription of FOXO1, leading to hyperglycaemia.^92^

Pathological concentrations of TMAO are upstream factors that activate the proinflammatory phenotype of astrocytes, impair their aerobic glycolytic metabolic plasticity, cause abnormalities in brain energy metabolism, and result in cognitive dysfunction. The 27‐month‐old mice had higher concentrations of TMAO in their plasma and brains, performed worse on new object recognition tests, and were associated with higher proinflammatory cytokine and astrocyte activation markers. Primary human astrocytes co‐cultured with TMAO exhibited morphological proliferation and hypertrophy, as well as increased reactive activation of LCN2 and CD44 protein markers.^93^ TMAO promotes microglia and astrocyte activation in mice with intracerebral hemorrhage and promotes a cellular inflammatory response.^94^ TMAO exacerbates ischemic nerve damage by activating astrocytes and forming glial scars via the Smurf2/ALK5 axis.^95^ In addition, chronic low‐dose long‐term exposure to TMAO protected the BBB from inflammatory challenges and preserves cognitive function. LPS exposure was associated with a significant decrease in endothelial GFAP+ astrocytes and IBA1+ microglia, which was effectively reversed with TMAO treatment.^96^

### 4.3 Aryl hydrocarbon receptors ligands

The microbial metabolism of dietary tryptophan can be decomposed into various biologically active molecules that bind directly to AhR as hydrocarbon receptor ligands. Through the gut microbiota, tryptophan metabolites may also affect CNS inflammation and contribute to neuropsychiatric disorders. As diabetes severity increases in the PREDIMED cohort, plasma tryptophan levels are likely to rise first and then deplete.^97^ The AhR pathway promotes microglia to produce TGF‐α and VEGF‐B to indirectly regulate the astrocyte transcriptional program. Microglia TGF‐α exerts its neuroprotective function through the ErbB1 receptor, promoting beneficial astrocyte activity. VEGF‐B triggers vascular endothelial growth factor receptor 1 (Flt‐1) signaling in the astrocyte.^90^ Conversely, Type I interferons (IFN‐I) signaling in combination with gut microbiota metabolites derived from dietary tryptophan activates AhR in astrocytes. Interferon alpha receptor 1 (IFNAR1‐1) plays an important role in anti‐neuroinflammation and preventing neurodegeneration.^98^

## 5 GUT MICROBIOTA‐ASTROCYTE AXIS CONNECTS T2DM WITH COGNITIVE DYSFUNCTION

**FIGURE 2:**
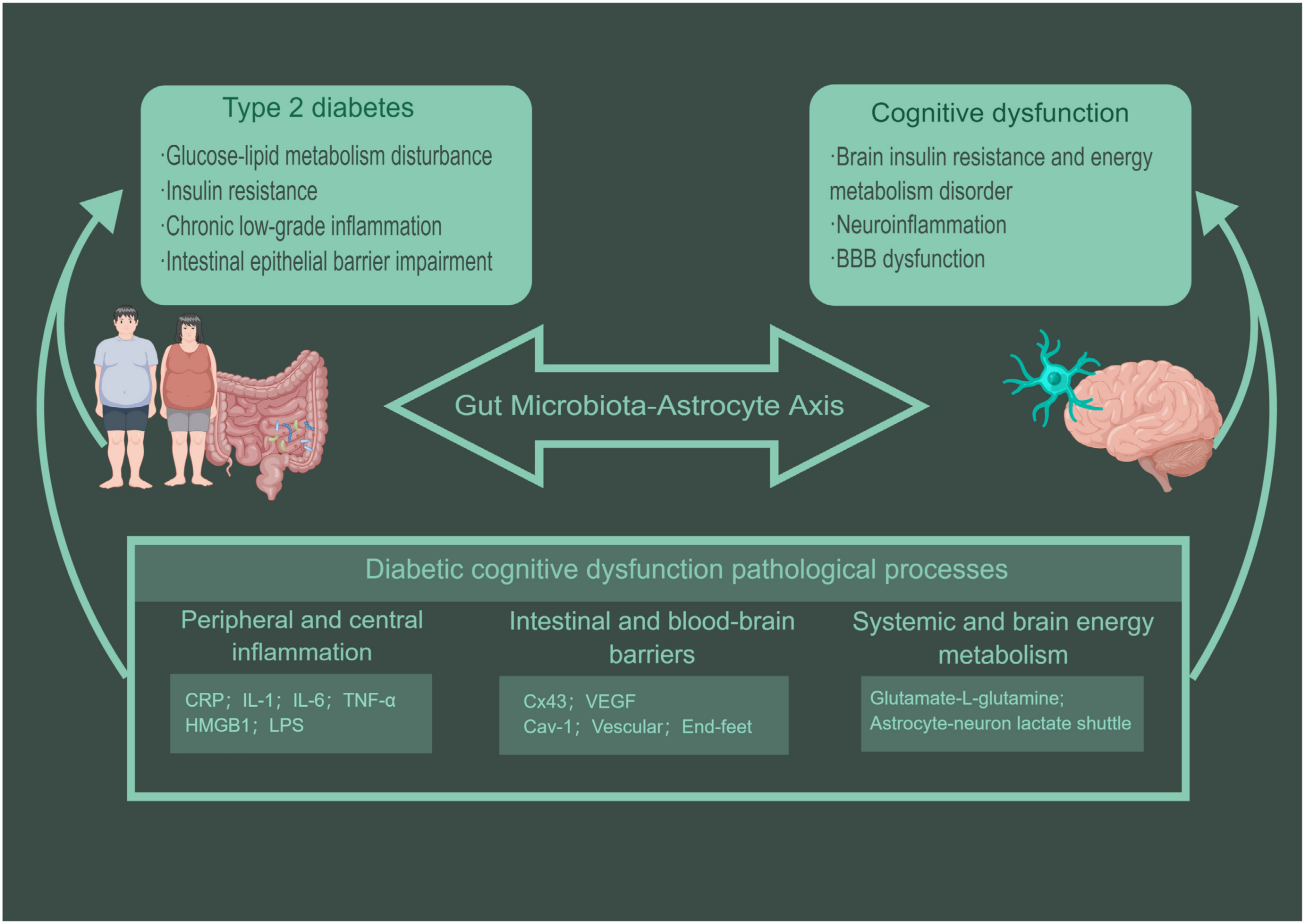
The “gut microbiota‐astrocyte” axis is coupled to the pathogenesis of cognitive dysfunction secondary to type 2 diabetes mellitus. Gut microbes and astrocytes are critical factors in the gut‐brain axis, leading to diabetic cognitive dysfunction through peripheral and central inflammation, gut and blood–brain barriers, and systemic and brain energy metabolism. Gut microbiota and its metabolites as upstream drivers of astrocytic activation. The reactive astrocytes' morphology and function changes result in blood–brain barrier injury, neuroinflammation, and brain energy metabolic disorder.

Diabetes‐related cognitive impairment is secondary to metabolic disturbances via the gut‐brain axis. Gut microbiota disturbance is the key link of metabolic inflammation, immune barrier damage, and energy metabolism. The astrocyte is an important regulator of the immune and metabolic balance of the brain. Gut microbiota, which drives astrocyte activation via neuroimmune pathways, is a target for the treatment and diagnosis of cognitive impairment in diabetes (Figure 2).

### 5.1 Gut microbiota and astrocyte co‐regulate central and peripheral inflammation

Chronic low‐grade inflammation is a hallmark of type 2 diabetes, leading to impaired β cell islet structure and function, inducing hepatic IR, and impairing glucose tolerance by blocking glucose‐stimulated insulin secretion (GSIS). Through dysbiosis of the gut microbiota, pathogen‐associated molecular patterns, damage‐associated and microbial‐associated patterns enter the circulatory system, inducing systemic inflammation and immune responses.^68^, ^99^ A cell wall component of gram‐negative bacteria, lipopolysaccharide (LPS), has been reported to be a source of metabolic inflammation. By activating macrophages through the inflammatory cytokine TLR4‐MyD88 pathway, LPS induces macrophages, dendritic cells, and other inflammation‐causing cells to form a worsened inflammatory microenvironment in metabolic tissues.^99^, ^100^

As a relatively immune‐privileged site, the neuroinflammation of the brain mostly results from the stimulation of circulating and gut‐derived inflammatory factors. The central recruitment and migration of metabolic inflammatory factors across the BBB promote astrocyte activation and transition to a responsive proinflammatory phenotype. It enhances inflammatory signaling and exacerbates neurodegeneration.^101^

Systemic chronic low‐grade inflammation is a common pathological mechanism of diabetes and dementia and is an important factor in accelerating central nerve damage in diabetes.^102^ Chronic inflammation within the peripheral and central nervous systems underlies cognitive dysfunction associated with metabolic syndrome.^103^ A prospective cohort study showed that plasma levels of C‐reactive protein (CRP), interleukin‐1 (IL‐1), interleukin‐6 (IL‐6), and tumor necrosis factor (TNF‐α) are associated with cognitive difficulties in older adults with T2DM.^104^ In a meta‐analysis of 40 clinical studies, elevated IL‐6, CRP, SVCAM‐1, and AGEs levels were suggested in cognitively impaired T2DM patients.^105^ Besides these peripheral markers of inflammation, CNS inflammation is also present in obese and T2DM patients, which manifests as neural immune cell infiltration, microglia, and astrocyte activation.

Astrocyte activation is a significant feature of neuroinflammation. Activated astrocyte gene expression switches to a proinflammatory and cytotoxic state, producing a variety of proinflammatory molecules, including cytokines, chemokines, complement factors, and ROS.^97^ The synthesis of LacCer in astrocytes promotes CNS‐infiltrating monocytes and microglia.^106^ The metabesity factor HMG20A is increased under mild inflammation induced by obesity and IR, inducing reactive astrocyte hyperplasia, protecting neurons from metabolic stress, and re‐establishing glucose homeostasis.^107^ Regulating COX‐mediated oxylipin synthesis in astrocytes as a novel potential target in the treatment of hyperglycemia‐associated brain injury.^108^

The Proinflammatory cytokine produced by dysbacteriosis is an important immune signal that directly activates astrocytes. The db/db mice have significantly increased escape latency at 6, 18, and 26 weeks of age, and their senescence‐associated cognitive decline is associated with the gut microbiome.^109^, ^110^ Phulwani et al.^111^ have shown that LPS induces astrocyte TLR2 via TNF‐α and NF‐κB pathways. Fecal microbiota transplantation (FMT)^112^ and vancomycin^113^ can inhibit the TLR4/TNF‐α and alleviate astrocyte‐related neuroinflammation. FMT treatment was involved in reducing depressive behavior by inhibiting astrocyte dysfunction in CIRCHIPK2‐expressing good mice.^114^ Recent studies have shown that gut microbes regulate the expression of Interferon‐γ in meningeal NK cells, thereby promoting the expression of astrocyte TRAIL, limiting CNS inflammation through induction of T‐cell apoptosis by Lamp1+ TRAIL+ astrocyte.^115^

### 5.2 Gut microbiota and astrocyte are critical hubs to the intestinal and blood–brain barrier

The TJs of the BBB are similar to those of the intestinal immune barrier, including Claudin‐5, occulin, and ZO‐1. An energetically favorable change in the gut barrier and BBB is an important pathway for interaction between the gut‐brain axis. The results showed that BBB dysfunction preceded early biomarkers of cognitive decline other than the accumulation of amyloid and tau proteins.^116^ Increased BBB permeability is one of the key pathologies of cognitive impairment in diabetes.^117^, ^118^ The accumulation of lipid peroxidation by‐products, advanced glycation end products, and mitochondrial superoxide production in diabetes mellitus, causing neovascularization abnormalities and increased capillary density in the CNS.

Blood–brain barrier damage varies in different types of diabetic animals. Increased BBB permeability in more than 84% of brain regions was found in BBZDR/WOR rats, whereas no significant changes were observed in the cerebellum and midbrain.^119^ In STZ‐induced diabetic rats, BBB permeability to small molecules gradually increased over a 28 to 90‐day period, and these changes were mainly observed in the midbrain, basal ganglia, cortex, and hippocampal regions.^120^ In the HFD diet‐induced model of obese T2DM rats, BBB damage predates cognitive impairment and occurs predominantly in the hypothalamus and hippocampus, and continues to deteriorate with the course of the disease.^121^ High‐fat and high‐fructose (HFHF) diet‐induced prediabetes mice experienced significant cognitive deterioration, accompanied by the permeability of the BBB and enhanced neuroinflammation in the cortex and hippocampus.^56^

Astrocyte is required for the maintenance of BBB integrity in the adult brain, and BBB regulators secreted by other cell types are insufficient to compensate for the loss of astrocyte.^122^ In addition to its role in vascularization, astrocytes exert synergic action with endothelial cells via paracrine and extracellular vesicle pathways to regulate TJ protein expression to control BBB integrity.^123^ For instance, astrocyte‐derived Wnt maintains the activity of Wnt/β‐catenin, thereby controlling Cav‐1 expression, vesicle abundance, and terminal integrity of the foot in NVU cells.^124^ During sustained inflammation, microglia phagocytose astrocytic endfeet and impair BBB function.^125^ Impaired BBB is accompanied by elevated GFAP and serves as a biomarker of cognitive impairment in diabetes.^126^ The co‐culture system of astrocyte and cerebral microvascular (CMEC) suggests that high glucose concentrations alter the expression of astrocyte Cx43 and increase VEGF secretion, resulting in impaired CMEC barrier properties.^127^

Gut microbiota‐associated metabolites promote peripheral immune cells to alter the structural integrity of the BBB.^128^ Gut microbiota‐BBB communication begins during pregnancy and spreads throughout life. Germ‐free mice showed increased BBB permeability and a consistent reduction in TJ protein expressions such as occulin and Claudin‐5 after birth and adulthood.^129^ Dysregulation of gut metabolites and disruption of the intestinal barrier caused by microbial perturbations promote the entry of deleterious metabolites into the circulatory system. Yu et al.^57^ showed that intestinal dysbiosis caused by a high‐fructose diet (HFrD) reduces SCFAs and damages the intestinal epithelial barrier, thus promoting astrocyte activation and BBB damage. In mouse models of obesity and diabetes, hyperglycemia entrains the permeability of the intestinal barrier. High glucose levels lead to retrograde glucose transport to intestinal epithelial cells via GLUT2, which subsequently alters intracellular glucose metabolism and transcriptional reprogramming, altering intestinal mucosal compactness and adhesion junction integrity.^130^ Microbiome and altered intestinal permeability increase circulating high mobility group protein 1 (HMGB1) and LPS, leading to systemic inflammation and disruption of the BBB.^131^ HMGB1 induces the production of proinflammatory cytokines and promotes neuroinflammation by activating TLR4 and RAGE in the astrocyte. Astrocyte's endfeet swelling, detachment from the basement membrane, and opening of the TJ between endothelial cells were strongly inhibited by the anti‐HMGB1 monoclonal antibody.^132^ The induction of astrocyte proliferation and activation by LPS promotes high expression of proinflammatory and cytotoxic genes, which may be related to BBB destruction.^133^

### 5.3 The gut microbiota‐astrocyte axis couples systemic and brain energy metabolism disorders

The brain uses glucose as its primary energy substrate, consuming 20 percent of the body's glucose and oxygen, to support the energy‐dependent synaptic activity of neurons. Brain energy metabolism decline is a hallmark event of cognitive decline, restoring energy metabolism as a novel therapeutic Neurodegeneration.^134^

Blood glucose fluctuation, frequent hypoglycemia events, IR, and other metabolic disorders are all risk factors for regional energy metabolism decline in the brain. Diabetes has been found in existing studies to reduce cerebral glucose utilization and cause nerve damage, possibly by altering BBB glucose transport uptake, neurotransmitter metabolism, and the ability to regulate cerebral blood flow.^135^, ^136^ According to a study including 323 adults with prediabetes, hyperglycemia was negatively associated with 18F‐FDG uptake in the prewedge lobe and occipital cortex.^104^ The brain is inefficient at energy acquisition after glucose loading in obese men, which could be due to abnormalities in glucose transport across the BBB or downregulation of energy synthesis during mitochondrial oxidation.^137^

The adaptive changes in brain energy metabolism were also observed in different animal models of diabetes. A recent multi‐omics analysis in 4‐month‐old db/db mice of cognitive impairment has identified disturbances in cerebral and circulatory mitochondrial metabolism.^138^ Huang et al.^139^ found that 26‐week‐old db/db mice had significantly reduced mitochondrial function and ATP content in the hippocampus. Andersen et al.^140^ found glucose hypometabolism in the cortex and hippocampal slices of db/db mice. The hippocampus showed enhanced ketone metabolism, and mitochondria in the cerebral cortex showed enhanced OXPHOS. These metabolic changes may be adaptive changes associated with low brain energy metabolism.

Astrocytes generate abundant mitochondrial reactive oxygen species, including lactic acid and serine, during glycolysis, which is coupled to neuronal OXPHOS to maintain brain energy requirements and relieve oxidative stress, and regulate the activity of neurotransmitter receptor.^39^ Astrocytes' glucose uptake and glycolytic plasticity play an essential role in maintaining brain energy metabolic balance. Astrocyte metabolic disorders have been observed in various animal models of diabetes. Girault et al.^141^ examined GK rats using ^13^C magnetic resonance spectroscopy and found that T2D impairs glutamate‐L‐Glutamine circuits in the brain between neurons and astrocytes, increasing the rate of TCA astrocytes.

Biological communication between the gut and brain is mediated by the gut microbiome, regulating the system and cerebral energy balance. C57BL/6J mice treated with HFD antibiotics altered the gut microbiota and multiple metabolites and improved insulin signaling and energy metabolism in the brain.^142^ Intermittent fasting (IF) can reconstitute the gut microbiota and metabolites of db/db mice, which in turn improves mitochondrial metabolism in the hippocampus and enhances genes associated with the OXPHOS pathway.^143^ *Akkermansia muciniphila* CIP107961 and environmental enrichment has been shown to reverse the metabolic abnormalities in the brain induced by the high‐fat, high‐cholesterol diet.^144^

Gut microbiota drives astrocyte activation along with metabolic switching, which in turn improves brain energy metabolism. Gut microbiota promotes the expression of PFKFB3 and ATP1A2, key proteins of hippocampal ANLS.^145^ STZ‐induced diabetic SD rats have cognitive impairment and intestinal flora disturbance. Disturbances in Glu/GABA‐Gln cycling and astrocyte energy metabolism in the rat hippocampus are thought to be associated with changes in *Clostridium_sensu_stricto_1*, *Romboutsia* and *Turicibacter*.^146^

## 6 THERAPEUTIC STRATEGIES OF GUT MICROBIOTA AND ASTROCYTE

Clinical guidelines on DCD emphasize the management of glucose and other metabolic homeostasis, reducing glucose fluctuations and avoiding hypoglycemic events.^147^ However, the benefits and mechanisms of anti‐diabetic drugs for DCD remain unclear. Cholinesterase inhibitors improve some dementia symptoms but fail to reverse the ongoing deterioration of cognitive impairment. The overlapping risk factors and the close pathophysiological relationship between diabetes and cognitive impairment have prompted interest in the role of anti‐diabetic drugs for cognition. There have been many excellent reviews suggesting the effects of sodium‐glucose co‐transporter 2 (SGLT2),^148^ dipeptidyl peptidase‐4 (DPP‐4) inhibitors,^149^ metformin,^150^ and glucagon‐like peptide‐1 receptor (GLP‐1R) agonist^151^ on cognitive function. But conflicting and divergent findings remain between the current studies, and the benefits and mechanisms of anti‐diabetic drugs on DCD remain unclear.^152^

Preclinical studies have observed that many anti‐diabetic drugs drive astrocyte phenotypic transformation. IR deficiency in astrocytes leads to impaired glucose tolerance and increases anxious‐depressive behavior. Intranasal insulin administration improves cognitive function.^153^ Astrocytes express IR and GLP‐1R, which activation affects brain glucose uptake and neurocognitive function. As an intestinal hormone, GLP‐1R agonists selectively block Aβ protein‐induced microglia activation and inhibits astrocyte‐responsive activation.^154^ GLP‐1R agonists also reduce astrocyte‐derived activators to protect the BBB and inhibit neuroinflammation.^155^ In addition, thiazolidinediones act as peroxisome proliferator‐activated receptor‐γ (PPARγ) agonists and exert neuroprotective effects by inhibiting GFAP expression and morphological proliferation of astrocytes.^156^, ^157^

Currently, new drug developments such as the seaweed derivative sodium oligomannate (GV‐971), have gone beyond neuronal centers and act by targeting the gut‐brain axis.^158^ Based on the broad utility of astrocytes and gut microbiota in neuroendocrinology, we take a step back and discuss those dietary interventions and natural compounds with considerable therapeutic potential.

### 6.1 Dietary interventions and probiotics

Restructuring the dietary structure is a direct way to modulate the gut microbiota and explore its effects. Intensive lifestyle interventions have been recognized as an essential clinical approach to reversing the course of diabetes and delaying the development of complications.^159^ Researchers' interest in nutritional psychiatry has driven many studies of specific dietary approaches such as the ketogenic diet,^160^ the Mediterranean diet,^161^ and Intermittent fasting^143^ to improve neurocognitive function. Genomic and proteomic sequencing reveals extensive immune and metabolic shifts in arcuate nucleus astrocytes in response to a high‐fat, high‐sugar diet.^162^ The ketogenic diet affects the metabolic plasticity of neurons and astrocytes,^163^ and research has shown that gut microbiota is necessary and sufficient for the neuroprotective effects.^164^ The calorie‐restricted diet reduces astrocyte glycolysis thereby limiting neuroinflammation.^165^, ^166^

Probiotics and synbiotics have been proposed as dietary supplements to intervene in metabolic disorders and cognitive impairment. Several research studies in vitro and in vivo have demonstrated that intake of probiotics can protect the intestinal mucosal barrier and promote the release of beneficial intestinal hormones and gut microbiota metabolites in patients with diabetes, thereby improving glycemic control and insulin resistance.^167^, ^168^ Probiotic supplementation increased the ratio of *Lactobacillus*/*Clostridium* and *Lactobacillus*/*Bacteroidetes*, promoted the BDNF/TrkB/CREB signaling pathway in diabetic rats, decreased the level of neuronal apoptosis, and effectively reversed the synaptic long‐time enhancement.^169^, ^170^

### 6.2 Traditional herbals and their active ingredients

Herbs and their active ingredients are potential treasures for targeting gut microflora dysbiosis and thereby improving cognitive function. A wealth of contemporary evidence supports its important role in preventing diabetic complications.^171^, ^172^ With the advancement of multi‐omics and genome sequencing technologies, in‐depth biological mechanisms have elaborated the holistic systemic concept of governance and multi‐targeted efficacy of TCM.^173^, ^174^ Focusing on the “gut microbiota‐astrocyte” axis, we screened the five most potentially therapeutic herbs and their derivatives. Ginsenosides are ginseng's main active pharmacological components.

Ginsenoside Rh4 inhibits astrocyte overactivation by promoting the enrichment of beneficial gut microbiota and increasing SCFAs content, thereby reducing hippocampal neuronal apoptosis and synaptic structural damage.^175^

*Astragalus* polysaccharide (APP) possesses hypoglycemic and cognitive protective effects on both db/db mice^176^ and STZ‐induced diabetic models of rats.^177^ APP increases the diversity of the gut microbiota, inhibits the potential intestinal pathogen *Shigella*, and enriches the beneficial bacteria *Homococcus* and *Lactobacillu*s.^178^ APP improves insulin resistance status in metabolically stressed mice and reduces astrocyte proliferation and activation around neuronal amyloid plaques.^179^

Berberine (BBR) is characterized by low oral utilization and significant regulation in the intestinal microflora. It has been found in many plants, such as *Coptis chinensis* Franch and *Phellodendron chinense* Schneid.^180^ BBR reduced hyperglycemia in diabetic rats while reducing oxidative stress in the hippocampus and preventing excessive activation of GFAP.^181^, ^182^

Curcumin and its analogs attenuate DCD by modulating inflammation and oxidative stress, and reduce aberrantly activated astrocytes in the hippocampus.^183^, ^184^ Curcumin reverses gut microbiota dysbiosis in diabetic rats, and increases *Bacteroides* and *Bifidobacterium* but inhibits *Enterobacteriaceae* and *Thicketella phylum*.^185^ In vitro studies have shown that curcumin modulates the binding of endogenous ligands to AhR, promotes AhR activation, and decreases LPS‐induced NF‐κB activation, thereby regulating inflammatory astrocyte proliferation.^186^ In addition, herbal active substances such as forsythoside B, rhubarb phenol, chrysin, resveratrol, and paeoniflorin have the effect of ameliorating DCD over gut microbiota and astrocytes, but direct and complete research evidence is still lacking.

Overall, the study of the gut‐brain axis in traditional medicine still needs to shift from analyzing correlations to establishing cause‐effect relationships. Observations on astrocytes have been mostly limited to neuroinflammation. Further enrichment of the modulatory effects of herbs on astrocyte phenotypes will help to uncover potential treatment of DCD and provide insight into the clinical efficacy of traditional herbals.

## 7 CONCLUSION

Though there is much evidence for cognitive impairment in patients with diabetes, the pathogenesis of DCD as a complication of diabetes is unclear and it is difficult to differentiate DCD from AD. Out of the multiple pathological factors, gut microbiota and its metabolites are important mediators to couple visceral and central environment, through the gut‐brain axis neuroimmune pathway, the unique two‐sided relationship between metabolic health and cognitive mind is highlighted. Unique anatomical and functional characteristics of astrocytes serve as intermediary glia in contact with circulating substances and brain microenvironment, maintaining immune and metabolic brain homeostasis and providing a continuous supply of energy‐dependent neuronal activity. With the development of genomics and cell sequencing technology, “gut microbiota‐astrocyte” axis research using the concept of system biology will help to deepen the understanding of the pathological mechanism of metabolic cognitive disorders, to explore an accurate and comprehensive treatment plan.

## AUTHOR CONTRIBUTIONS

All authors read, revised, and approved the final manuscript.

## CONFLICT OF INTEREST

The authors declare that they have no competing interests.

## Data Availability

Data sharing is not applicable to this article as no new data were created or analyzed in this study.

## References

[1] American Diabetes Association . 12. Older adults: standards of medical care in diabetes‐2021. Diabetes Care. 2021;44(Suppl 1):S168‐S179. 33298423 10.2337/dc21-S012

[2] Ott A , Stolk RP , van Harskamp F , Pols HA , Hofman A , Breteler MM . Diabetes mellitus and the risk of dementia: the Rotterdam study. Neurology. 1999;53(9):1937‐1942. 10599761 10.1212/wnl.53.9.1937

[3] Chatterjee S , Peters SA , Woodward M , et al. Type 2 diabetes as a risk factor for dementia in women compared with men: a pooled analysis of 2.3 million people comprising more than 100,000 cases of dementia. Diabetes Care. 2016;39(2):300‐307. 26681727 10.2337/dc15-1588 PMC4722942

[4] Biessels GJ , Staekenborg S , Brunner E , Brayne C , Scheltens P . Risk of dementia in diabetes mellitus: a systematic review. Lancet Neurol. 2006;5(1):64‐74. 16361024 10.1016/S1474-4422(05)70284-2

[5] Xue M , Xu W , Ou YN , et al. Diabetes mellitus and risks of cognitive impairment and dementia: a systematic review and meta‐analysis of 144 prospective studies. Ageing Res Rev. 2019;55:100944. 31430566 10.1016/j.arr.2019.100944

[6] Pelimanni E , Jehkonen M . Type 2 diabetes and cognitive functions in middle age: a meta‐analysis. J Int Neuropsychol Soc. 2019;25(2):215‐229. 30575498 10.1017/S1355617718001042

[7] Biessels GJ , Deary IJ , Ryan CM . Cognition and diabetes: a lifespan perspective. Lancet Neurol. 2008;7(2):184‐190. 18207116 10.1016/S1474-4422(08)70021-8

[8] Brady CC , Vannest JJ , Dolan LM , et al. Obese adolescents with type 2 diabetes perform worse than controls on cognitive and behavioral assessments. Pediatr Diabetes. 2017;18(4):297‐303. 27028236 10.1111/pedi.12383

[9] Srikanth V , Sinclair AJ , Hill‐Briggs F , Moran C , Biessels GJ . Type 2 diabetes and cognitive dysfunction‐towards effective management of both comorbidities. Lancet Diabetes Endocrinol. 2020;8(6):535‐545. 32445740 10.1016/S2213-8587(20)30118-2

[10] van den Berg E , de Craen AJ , Biessels GJ , Gussekloo J , Westendorp RG . The impact of diabetes mellitus on cognitive decline in the oldest of the old: a prospective population‐based study. Diabetologia. 2006;49(9):2015‐2023. 16804671 10.1007/s00125-006-0333-1

[11] Arvanitakis Z , Schneider JA , Wilson RS , et al. Diabetes is related to cerebral infarction but not to AD pathology in older persons. Neurology. 2006;67(11):1960‐1965. 17159101 10.1212/01.wnl.0000247053.45483.4e

[12] Dos Santos Matioli MNP , Suemoto CK , Rodriguez RD , et al. Diabetes is not associated with Alzheimer's disease neuropathology. J Alzheimer's Dis. 2017;60(3):1035‐1043. 28984587 10.3233/JAD-170179 PMC5892204

[13] Chornenkyy Y , Wang WX , Wei A , Nelson PT . Alzheimer's disease and type 2 diabetes mellitus are distinct diseases with potential overlapping metabolic dysfunction upstream of observed cognitive decline. Brain Pathol. 2019;29(1):3‐17. 10.1111/bpa.12655 PMC6427919 30106209

[14] Cardoso S , Moreira PI . Diabesity and brain disturbances: a metabolic perspective. Mol Aspects Med. 2019;66:71‐79. 30321556 10.1016/j.mam.2018.10.002

[15] Kullmann S , Heni M , Hallschmid M , Fritsche A , Preissl H , Häring HU . Brain insulin resistance at the crossroads of metabolic and cognitive disorders in humans. Physiol Rev. 2016;96(4):1169‐1209. 27489306 10.1152/physrev.00032.2015

[16] Chen YC , Jiao Y , Cui Y , et al. Aberrant brain functional connectivity related to insulin resistance in type 2 diabetes: a resting‐state fMRI study. Diabetes Care. 2014;37(6):1689‐1696. 24658392 10.2337/dc13-2127

[17] van Sloten TT , Sedaghat S , Carnethon MR , Launer LJ , Stehouwer CDA . Cerebral microvascular complications of type 2 diabetes: stroke, cognitive dysfunction, and depression. Lancet Diabetes Endocrinol. 2020;8(4):325‐336. 32135131 10.1016/S2213-8587(19)30405-X PMC11044807

[18] Wardlaw JM , Smith EE , Biessels GJ , et al. Neuroimaging standards for research into small vessel disease and its contribution to ageing and neurodegeneration. Lancet Neurol. 2013;12(8):822‐838. 23867200 10.1016/S1474-4422(13)70124-8 PMC3714437

[19] Pugazhenthi S , Qin L , Reddy PH . Common neurodegenerative pathways in obesity, diabetes, and Alzheimer's disease. Biochim Biophys Acta Mol Basis Dis. 2017;1863(5):1037‐1045. 27156888 10.1016/j.bbadis.2016.04.017 PMC5344771

[20] Biessels GJ , Reijmer YD . Brain changes underlying cognitive dysfunction in diabetes: what can we learn from MRI? Diabetes. 2014;63(7):2244‐2252. 24931032 10.2337/db14-0348

[21] Akisaki T , Sakurai T , Takata T , et al. Cognitive dysfunction associates with white matter hyperintensities and subcortical atrophy on magnetic resonance imaging of the elderly diabetes mellitus Japanese elderly diabetes intervention trial (J‐EDIT). Diabetes Metab Res Rev. 2006;22(5):376‐384. 16506272 10.1002/dmrr.632

[22] Zhang T , Shaw M , Cherbuin N . Association between type 2 diabetes mellitus and brain atrophy: a meta‐analysis. Diabetes Metab J. 2022;46:781‐802. 35255549 10.4093/dmj.2021.0189 PMC9532183

[23] Callisaya ML , Beare R , Moran C , Phan T , Wang W , Srikanth VK . Type 2 diabetes mellitus, brain atrophy and cognitive decline in older people: a longitudinal study. Diabetologia. 2019;62(3):448‐458. 30547230 10.1007/s00125-018-4778-9

[24] Li W , Risacher SL , Huang E , Saykin AJ . Type 2 diabetes mellitus is associated with brain atrophy and hypometabolism in the ADNI cohort. Neurology. 2016;87(6):595‐600. 27385744 10.1212/WNL.0000000000002950 PMC4977372

[25] Moran C , Phan TG , Chen J , et al. Brain atrophy in type 2 diabetes: regional distribution and influence on cognition. Diabetes Care. 2013;36(12):4036‐4042. 23939539 10.2337/dc13-0143 PMC3836136

[26] Sanjari Moghaddam H , Ghazi Sherbaf F , Aarabi MH . Brain microstructural abnormalities in type 2 diabetes mellitus: a systematic review of diffusion tensor imaging studies. Front Neuroendocrinol. 2019;55:100782. 31401292 10.1016/j.yfrne.2019.100782

[27] Huang L , Zhang Q , Tang T , et al. Abnormalities of brain white matter in type 2 diabetes mellitus: a meta‐analysis of diffusion tensor imaging. Front Aging Neurosci. 2021;13:693890. 34421572 10.3389/fnagi.2021.693890 PMC8378805

[28] Reijmer YD , Leemans A , Brundel M , Kappelle LJ , Biessels GJ . Disruption of the cerebral white matter network is related to slowing of information processing speed in patients with type 2 diabetes. Diabetes. 2013;62(6):2112‐2115. 23349494 10.2337/db12-1644 PMC3661620

[29] Musen G , Jacobson AM , Bolo NR , et al. Resting‐state brain functional connectivity is altered in type 2 diabetes. Diabetes. 2012;61(9):2375‐2379. 22664957 10.2337/db11-1669 PMC3425418

[30] LeRoith D , Biessels GJ , Braithwaite SS , et al. Treatment of diabetes in older adults: an Endocrine Society* clinical practice guideline. J Clin Endocrinol Metab. 2019;104(5):1520‐1574. 30903688 10.1210/jc.2019-00198 PMC7271968

[31] Committee Report: Glycemic targets for elderly patients with diabetes: Japan diabetes society (JDS)/Japan geriatrics society (JGS) joint committee on improving Care for Elderly Patients with diabetes. J Diabetes Investig. 2017;8(1):126‐128. 10.1111/jdi.12599 PMC5217949 28054465

[32] Biessels GJ , Whitmer RA . Cognitive dysfunction in diabetes: how to implement emerging guidelines. Diabetologia. 2020;63(1):3‐9. 31420699 10.1007/s00125-019-04977-9 PMC6890615

[33] Geijselaers SLC , Sep SJS , Stehouwer CDA , Biessels GJ . Glucose regulation, cognition, and brain MRI in type 2 diabetes: a systematic review. Lancet Diabetes Endocrinol. 2015;3(1):75‐89. 25163604 10.1016/S2213-8587(14)70148-2

[34] Ghaisas S , Maher J , Kanthasamy A . Gut microbiome in health and disease: linking the microbiome‐gut‐brain axis and environmental factors in the pathogenesis of systemic and neurodegenerative diseases. Pharmacol Ther. 2016;158:52‐62. 26627987 10.1016/j.pharmthera.2015.11.012 PMC4747781

[35] Zhao YF , Wei DN , Tang Y . Gut microbiota regulate astrocytic functions in the brain: possible therapeutic consequences. Curr Neuropharmacol. 2021;19(8):1354‐1366. 33588733 10.2174/1570159X19666210215123239 PMC8719287

[36] Johnson ECB , Dammer EB , Duong DM , et al. Large‐scale proteomic analysis of Alzheimer's disease brain and cerebrospinal fluid reveals early changes in energy metabolism associated with microglia and astrocyte activation. Nat Med. 2020;26(5):769‐780. 32284590 10.1038/s41591-020-0815-6 PMC7405761

[37] Allen NJ , Barres BA . Neuroscience: glia – more than just brain glue. Nature. 2009;457(7230):675‐677. 19194443 10.1038/457675a

[38] Horng S , Therattil A , Moyon S , et al. Astrocytic tight junctions control inflammatory CNS lesion pathogenesis. J Clin Invest. 2017;127(8):3136‐3151. 28737509 10.1172/JCI91301 PMC5531407

[39] Bonvento G , Bolaños JP . Astrocyte‐neuron metabolic cooperation shapes brain activity. Cell Metab. 2021;33(8):1546‐1564. 34348099 10.1016/j.cmet.2021.07.006

[40] LeMaistre JL , Anderson CM . Custom astrocyte‐mediated vasomotor responses to neuronal energy demand. Genome Biol. 2009;10(2):209. 19232077 10.1186/gb-2009-10-2-209 PMC2688276

[41] Cauli B , Hamel E . Brain perfusion and astrocytes. Trends Neurosci. 2018;41(7):409‐413. 29933772 10.1016/j.tins.2018.04.010

[42] Müller MS , Fouyssac M , Taylor CW . Effective glucose uptake by human astrocytes requires its sequestration in the endoplasmic reticulum by glucose‐6‐phosphatase‐β. Curr Biol. 2018;28(21):3481‐3486.e3484. 30415704 10.1016/j.cub.2018.08.060 PMC6224479

[43] Bélanger M , Allaman I , Magistretti PJ . Brain energy metabolism: focus on astrocyte‐neuron metabolic cooperation. Cell Metab. 2011;14(6):724‐738. 22152301 10.1016/j.cmet.2011.08.016

[44] Takahashi S . Neuroprotective function of high glycolytic activity in astrocytes: common roles in stroke and neurodegenerative diseases. Int J Mol Sci. 2021;22(12):6568. 34207355 10.3390/ijms22126568 PMC8234992

[45] Ameroso D , Meng A , Chen S , Felsted J , Dulla CG , Rios M . Astrocytic BDNF signaling within the ventromedial hypothalamus regulates energy homeostasis. Nat Metab. 2022;4(5):627‐643. 35501599 10.1038/s42255-022-00566-0 PMC9177635

[46] Cheung G , Bataveljic D , Visser J , et al. Physiological synaptic activity and recognition memory require astroglial glutamine. Nat Commun. 2022;13(1):753. 35136061 10.1038/s41467-022-28331-7 PMC8826940

[47] Andersen JV , Schousboe A , Verkhratsky A . Astrocyte energy and neurotransmitter metabolism in Alzheimer's disease: integration of the glutamate/GABA‐glutamine cycle. Prog Neurobiol. 2022;217:102331. 35872221 10.1016/j.pneurobio.2022.102331

[48] Suzuki A , Stern SA , Bozdagi O , et al. Astrocyte‐neuron lactate transport is required for long‐term memory formation. Cell. 2011;144(5):810‐823. 21376239 10.1016/j.cell.2011.02.018 PMC3073831

[49] Robin LM , Oliveira da Cruz JF , Langlais VC , et al. Astroglial CB(1) receptors determine synaptic D‐serine availability to enable recognition memory. Neuron. 2018;98(5):935‐944.e935. 29779943 10.1016/j.neuron.2018.04.034

[50] Lee JH , Kim JY , Noh S , et al. Astrocytes phagocytose adult hippocampal synapses for circuit homeostasis. Nature. 2021;590(7847):612‐617. 33361813 10.1038/s41586-020-03060-3

[51] Kol A , Adamsky A , Groysman M , Kreisel T , London M , Goshen I . Astrocytes contribute to remote memory formation by modulating hippocampal‐cortical communication during learning. Nat Neurosci. 2020;23(10):1229‐1239. 32747787 10.1038/s41593-020-0679-6 PMC7611962

[52] Zhang Y , Reichel JM , Han C , Zuniga‐Hertz JP , Cai D . Astrocytic process plasticity and IKKβ/NF‐κB in central control of blood glucose, blood pressure, and body weight. Cell Metab. 2017;25(5):1091‐1102.e1094. 28467927 10.1016/j.cmet.2017.04.002 PMC5576872

[53] García‐Cáceres C , Quarta C , Varela L , et al. Astrocytic insulin signaling couples brain glucose uptake with nutrient availability. Cell. 2016;166(4):867‐880. 27518562 10.1016/j.cell.2016.07.028 PMC8961449

[54] Rahman MH , Bhusal A , Kim JH , et al. Astrocytic pyruvate dehydrogenase kinase‐2 is involved in hypothalamic inflammation in mouse models of diabetes. Nat Commun. 2020;11(1):5906. 33219201 10.1038/s41467-020-19576-1 PMC7680139

[55] Wong DP , Chu JM , Hung VK , et al. Modulation of endoplasmic reticulum chaperone GRP78 by high glucose in hippocampus of streptozotocin‐induced diabetic mice and C6 astrocytic cells. Neurochem Int. 2013;63(6):551‐560. 24056253 10.1016/j.neuint.2013.09.010

[56] Takechi R , Lam V , Brook E , et al. Blood‐brain barrier dysfunction precedes cognitive decline and neurodegeneration in diabetic insulin resistant mouse model: an implication for causal link. Front Aging Neurosci. 2017;9:399. 29249964 10.3389/fnagi.2017.00399 PMC5717019

[57] Yu R , Wen S , Wang Q , et al. Mulberroside a repairs high fructose diet‐induced damage of intestinal epithelial and blood‐brain barriers in mice: a potential for preventing hippocampal neuroinflammatory injury. J Neurochem. 2021;157(6):1979‐1991. 33205422 10.1111/jnc.15242

[58] Zhang Z , Zhou H , Zhou J . Neuritin inhibits astrogliosis to ameliorate diabetic cognitive dysfunction. J Mol Endocrinol. 2021;66(4):259‐272. 33729996 10.1530/JME-20-0321 PMC8111324

[59] Hayden MR , Grant DG , Aroor AR , DeMarco VG . Empagliflozin ameliorates type 2 diabetes‐induced ultrastructural remodeling of the neurovascular unit and neuroglia in the female db/db mouse. Brain Sci. 2019;9(3):57. 30866531 10.3390/brainsci9030057 PMC6468773

[60] Shi S , Yin HJ , Li J , Wang L , Wang WP , Wang XL . Studies of pathology and pharmacology of diabetic encephalopathy with KK‐ay mouse model. CNS Neurosci Ther. 2020;26(3):332‐342. 31401815 10.1111/cns.13201 PMC7052806

[61] Nagayach A , Patro N , Patro I . Astrocytic and microglial response in experimentally induced diabetic rat brain. Metab Brain Dis. 2014;29(3):747‐761. 24833555 10.1007/s11011-014-9562-z

[62] Lechuga‐Sancho AM , Arroba AI , Frago LM , et al. Reduction in the number of astrocytes and their projections is associated with increased synaptic protein density in the hypothalamus of poorly controlled diabetic rats. Endocrinology. 2006;147(11):5314‐5324. 16873533 10.1210/en.2006-0766

[63] Nardin P , Zanotto C , Hansen F , et al. Peripheral levels of AGEs and astrocyte alterations in the hippocampus of STZ‐diabetic rats. Neurochem Res. 2016;41(8):2006‐2016. 27084774 10.1007/s11064-016-1912-2

[64] Lebed YV , Orlovsky MA , Nikonenko AG , Ushakova GA , Skibo GG . Early reaction of astroglial cells in rat hippocampus to streptozotocin‐induced diabetes. Neurosci Lett. 2008;444(2):181‐185. 18708122 10.1016/j.neulet.2008.07.094

[65] Lu J , Yang L , Xu Y , et al. The modulatory effect of motor cortex astrocytes on diabetic neuropathic pain. J Neurosci. 2021;41(24):5287‐5302. 33753547 10.1523/JNEUROSCI.2566-20.2021 PMC8211549

[66] Liu X , He J , Gao J , Xiao Z . Fluorocitrate and neurotropin confer analgesic effects on neuropathic pain in diabetic rats via inhibition of astrocyte activation in the periaqueductal gray. Neurosci Lett. 2022;768:136378. 34861344 10.1016/j.neulet.2021.136378

[67] Chu X , Zhou S , Sun R , et al. Chrysophanol relieves cognition deficits and neuronal loss through inhibition of inflammation in diabetic mice. Neurochem Res. 2018;43(4):972‐983. 29497904 10.1007/s11064-018-2503-1

[68] Gurung M , Li Z , You H , et al. Role of gut microbiota in type 2 diabetes pathophysiology. EBioMedicine. 2020;51:102590. 31901868 10.1016/j.ebiom.2019.11.051 PMC6948163

[69] Le Chatelier E , Nielsen T , Qin J , et al. Richness of human gut microbiome correlates with metabolic markers. Nature. 2013;500(7464):541‐546. 23985870 10.1038/nature12506

[70] Chen Z , Radjabzadeh D , Chen L , et al. Association of insulin resistance and type 2 diabetes with gut microbial diversity: a microbiome‐wide analysis from population studies. JAMA Netw Open. 2021;4(7):e2118811. 34323983 10.1001/jamanetworkopen.2021.18811 PMC8322996

[71] Alkasir R , Li J , Li X , Jin M , Zhu B . Human gut microbiota: the links with dementia development. Protein Cell. 2017;8(2):90‐102. 27866330 10.1007/s13238-016-0338-6 PMC5291774

[72] Meyer K , Lulla A , Debroy K , et al. Association of the gut microbiota with cognitive function in midlife. JAMA Netw Open. 2022;5(2):e2143941. 35133436 10.1001/jamanetworkopen.2021.43941 PMC8826173

[73] Zhang Y , Lu S , Yang Y , et al. The diversity of gut microbiota in type 2 diabetes with or without cognitive impairment. Aging Clin Exp Res. 2021;33(3):589‐601. 32301029 10.1007/s40520-020-01553-9

[74] Fung TC , Olson CA , Hsiao EY . Interactions between the microbiota, immune and nervous systems in health and disease. Nat Neurosci. 2017;20(2):145‐155. 28092661 10.1038/nn.4476 PMC6960010

[75] Sterka D Jr , Rati DM , Marriott I . Functional expression of NOD2, a novel pattern recognition receptor for bacterial motifs, in primary murine astrocytes. Glia. 2006;53(3):322‐330. 16265673 10.1002/glia.20286

[76] Li L , Acioglu C , Heary RF , Elkabes S . Role of astroglial toll‐like receptors (TLRs) in central nervous system infections, injury and neurodegenerative diseases. Brain Behav Immun. 2021;91:740‐755. 33039660 10.1016/j.bbi.2020.10.007 PMC7543714

[77] Dong Y , Benveniste EN . Immune function of astrocytes. Glia. 2001;36(2):180‐190. 11596126 10.1002/glia.1107

[78] Luo P , Lednovich K , Xu K , Nnyamah C , Layden BT , Xu P . Central and peripheral regulations mediated by short‐chain fatty acids on energy homeostasis. Transl Res. 2022;248:128‐150. 35688319 10.1016/j.trsl.2022.06.003 PMC12553404

[79] Spichak S , Donoso F , Moloney GM , et al. Microbially‐derived short‐chain fatty acids impact astrocyte gene expression in a sex‐specific manner. Brain Behav Immun Health. 2021;16:100318. 34589808 10.1016/j.bbih.2021.100318 PMC8474187

[80] Nie Q , Chen H , Hu J , Fan S , Nie S . Dietary compounds and traditional Chinese medicine ameliorate type 2 diabetes by modulating gut microbiota. Crit Rev Food Sci Nutr. 2019;59(6):848‐863. 30569745 10.1080/10408398.2018.1536646

[81] Cuervo‐Zanatta D , Syeda T , Sánchez‐Valle V , et al. Dietary fiber modulates the release of gut bacterial products preventing cognitive decline in an Alzheimer's mouse model. Cell Mol Neurobiol. 2022. 10.1007/s10571-022-01268-7 PMC11412426 35953741

[82] Wang C , Zheng D , Weng F , Jin Y , He L . Sodium butyrate ameliorates the cognitive impairment of Alzheimer's disease by regulating the metabolism of astrocytes. Psychopharmacology. 2022;239(1):215‐227. 34812899 10.1007/s00213-021-06025-0

[83] Natrus LV , Osadchuk YS , Lisakovska OO , Labudzinskyi DO , Klys YG , Chaikovsky YB . Effect of propionic acid on diabetes‐induced impairment of unfolded protein response signaling and astrocyte/microglia crosstalk in rat ventromedial nucleus of the hypothalamus. Neural Plast. 2022;2022:6404964. 35103058 10.1155/2022/6404964 PMC8800605

[84] Wyss MT , Magistretti PJ , Buck A , Weber B . Labeled acetate as a marker of astrocytic metabolism. J Cereb Blood Flow Metab. 2011;31(8):1668‐1674. 21654698 10.1038/jcbfm.2011.84 PMC3170955

[85] Kato H , Okuno T , Isohashi K , et al. Astrocyte metabolism in multiple sclerosis investigated by 1‐C‐11 acetate PET. J Cereb Blood Flow Metab. 2021;41(2):369‐379. 32169013 10.1177/0271678X20911469 PMC7812519

[86] Vogt NM , Romano KA , Darst BF , et al. The gut microbiota‐derived metabolite trimethylamine N‐oxide is elevated in Alzheimer's disease. Alzheimers Res Ther. 2018;10(1):124. 30579367 10.1186/s13195-018-0451-2 PMC6303862

[87] Connell E , Le Gall G , Pontifex MG , et al. Microbial‐derived metabolites as a risk factor of age‐related cognitive decline and dementia. Mol Neurodegener. 2022;17(1):43. 35715821 10.1186/s13024-022-00548-6 PMC9204954

[88] Barrea L , Annunziata G , Muscogiuri G , et al. Trimethylamine‐N‐oxide (TMAO) as novel potential biomarker of early predictors of metabolic syndrome. Nutrients. 2018;10(12):1971. 30551613 10.3390/nu10121971 PMC6316855

[89] Yoo W , Zieba JK , Foegeding NJ , et al. High‐fat diet‐induced colonocyte dysfunction escalates microbiota‐derived trimethylamine N‐oxide. Science (New York, NY). 2021;373(6556):813‐818. 10.1126/science.aba3683 PMC8506909 34385401

[90] Zhuang R , Ge X , Han L , et al. Gut microbe‐generated metabolite trimethylamine N‐oxide and the risk of diabetes: a systematic review and dose‐response meta‐analysis. Obes Rev. 2019;20(6):883‐894. 30868721 10.1111/obr.12843

[91] Miao J , Ling AV , Manthena PV , et al. Flavin‐containing monooxygenase 3 as a potential player in diabetes‐associated atherosclerosis. Nat Commun. 2015;6:6498. 25849138 10.1038/ncomms7498 PMC4391288

[92] Chen S , Henderson A , Petriello MC , et al. Trimethylamine N‐oxide binds and activates PERK to promote metabolic dysfunction. Cell Metab. 2019;30(6):1141‐1151.e1145. 31543404 10.1016/j.cmet.2019.08.021

[93] Brunt VE , LaRocca TJ , Bazzoni AE , et al. The gut microbiome‐derived metabolite trimethylamine N‐oxide modulates neuroinflammation and cognitive function with aging. GeroScience. 2021;43(1):377‐394. 32862276 10.1007/s11357-020-00257-2 PMC8050157

[94] Li C , Zhu L , Dai Y , et al. Diet‐induced high serum levels of trimethylamine‐N‐oxide enhance the cellular inflammatory response without exacerbating acute intracerebral hemorrhage injury in mice. Oxid Med Cell Longev. 2022;2022:1599747‐1599716. 35242275 10.1155/2022/1599747 PMC8886754

[95] Su H , Fan S , Zhang L , Qi H . TMAO aggregates neurological damage following ischemic stroke by promoting reactive astrocytosis and glial scar formation via the Smurf2/ALK5 Axis. Front Cell Neurosci. 2021;15:569424. 33815059 10.3389/fncel.2021.569424 PMC8012716

[96] Hoyles L , Pontifex MG , Rodriguez‐Ramiro I , et al. Regulation of blood‐brain barrier integrity by microbiome‐associated methylamines and cognition by trimethylamine N‐oxide. Microbiome. 2021;9(1):235. 34836554 10.1186/s40168-021-01181-z PMC8626999

[97] Yu E , Papandreou C , Ruiz‐Canela M , et al. Association of tryptophan metabolites with incident type 2 diabetes in the PREDIMED trial: a case‐cohort study. Clin Chem. 2018;64(8):1211‐1220. 29884676 10.1373/clinchem.2018.288720 PMC6218929

[98] Rothhammer V , Mascanfroni ID , Bunse L , et al. Type I interferons and microbial metabolites of tryptophan modulate astrocyte activity and central nervous system inflammation via the aryl hydrocarbon receptor. Nat Med. 2016;22(6):586‐597. 27158906 10.1038/nm.4106 PMC4899206

[99] Yang G , Wei J , Liu P , et al. Role of the gut microbiota in type 2 diabetes and related diseases. Metab Clin Exp. 2021;117:154712. 33497712 10.1016/j.metabol.2021.154712

[100] Scheithauer TPM , Rampanelli E , Nieuwdorp M , et al. Gut microbiota as a trigger for metabolic inflammation in obesity and type 2 diabetes. Front Immunol. 2020;11:571731. 33178196 10.3389/fimmu.2020.571731 PMC7596417

[101] Walker KA , Ficek BN , Westbrook R . Understanding the role of systemic inflammation in Alzheimer's disease. ACS Chem Neurosci. 2019;10(8):3340‐3342. 31241312 10.1021/acschemneuro.9b00333

[102] Dove A , Shang Y , Xu W , et al. The impact of diabetes on cognitive impairment and its progression to dementia. Alzheimer's Dement. 2021;17(11):1769‐1778. 34636485 10.1002/alz.12482

[103] Arnoriaga‐Rodríguez M , Fernández‐Real JM . Microbiota impacts on chronic inflammation and metabolic syndrome – related cognitive dysfunction. Rev Endocr Metab Disord. 2019;20(4):473‐480. 31884557 10.1007/s11154-019-09537-5

[104] Apostolova I , Lange C , Suppa P , et al. Impact of plasma glucose level on the pattern of brain FDG uptake and the predictive power of FDG PET in mild cognitive impairment. Eur J Nucl Med Mol Imaging. 2018;45(8):1417‐1422. 29502311 10.1007/s00259-018-3985-4

[105] Anita NZ , Zebarth J , Chan B , et al. Inflammatory markers in type 2 diabetes with vs. without cognitive impairment; a systematic review and meta‐analysis. Brain Behav Immun. 2022;100:55‐69. 34808290 10.1016/j.bbi.2021.11.005

[106] Mayo L , Trauger SA , Blain M , et al. Regulation of astrocyte activation by glycolipids drives chronic CNS inflammation. Nat Med. 2014;20(10):1147‐1156. 25216636 10.1038/nm.3681 PMC4255949

[107] Lorenzo PI , Martin Vazquez E , López‐Noriega L , et al. The metabesity factor HMG20A potentiates astrocyte survival and reactive astrogliosis preserving neuronal integrity. Theranostics. 2021;11(14):6983‐7004. 34093866 10.7150/thno.57237 PMC8171100

[108] Chistyakov DV , Goriainov SV , Astakhova AA , Sergeeva MG . High glucose shifts the oxylipin profiles in the astrocytes towards pro‐inflammatory states. Metabolites. 2021;11(5):311. 34068011 10.3390/metabo11050311 PMC8152232

[109] Zhang J , Zhang Y , Yuan Y , Liu L , Zhao Y , Wang X . Gut microbiota alteration is associated with cognitive deficits in genetically diabetic (Db/db) mice during aging. Front Aging Neurosci. 2021;13:815562. 35153726 10.3389/fnagi.2021.815562 PMC8826473

[110] Arranz AM , De Strooper B . The role of astroglia in Alzheimer's disease: pathophysiology and clinical implications. Lancet Neurol. 2019;18(4):406‐414. 30795987 10.1016/S1474-4422(18)30490-3

[111] Phulwani NK , Esen N , Syed MM , Kielian T . TLR2 expression in astrocytes is induced by TNF‐alpha‐ and NF‐kappa B‐dependent pathways. J. Immunol. 2008;181(6):3841‐3849. 18768838 10.4049/jimmunol.181.6.3841 PMC2649826

[112] Sun MF , Zhu YL , Zhou ZL , et al. Neuroprotective effects of fecal microbiota transplantation on MPTP‐induced Parkinson's disease mice: gut microbiota, glial reaction and TLR4/TNF‐α signaling pathway. Brain Behav Immun. 2018;70:48‐60. 29471030 10.1016/j.bbi.2018.02.005

[113] Cui C , Hong H , Shi Y , et al. Vancomycin pretreatment on MPTP‐induced Parkinson's disease mice exerts neuroprotection by suppressing inflammation both in brain and gut. J Neuroimmune Pharmacol. 2022. 10.1007/s11481-021-10047-y 35091889

[114] Zhang Y , Huang R , Cheng M , et al. Gut microbiota from NLRP3‐deficient mice ameliorates depressive‐like behaviors by regulating astrocyte dysfunction via circHIPK2. Microbiome. 2019;7(1):116. 31439031 10.1186/s40168-019-0733-3 PMC6706943

[115] Sanmarco LM , Wheeler MA , Gutiérrez‐Vázquez C , et al. Gut‐licensed IFNγ(+) NK cells drive LAMP1(+)TRAIL(+) anti‐inflammatory astrocytes. Nature. 2021;590(7846):473‐479. 33408417 10.1038/s41586-020-03116-4 PMC8039910

[116] Nation DA , Sweeney MD , Montagne A , et al. Blood‐brain barrier breakdown is an early biomarker of human cognitive dysfunction. Nat Med. 2019;25(2):270‐276. 30643288 10.1038/s41591-018-0297-y PMC6367058

[117] Bogush M , Heldt NA , Persidsky Y . Blood brain barrier injury in diabetes: unrecognized effects on brain and cognition. J Neuroimmune Pharmacol. 2017;12(4):593‐601. 28555373 10.1007/s11481-017-9752-7 PMC5693692

[118] Janelidze S , Hertze J , Nägga K , et al. Increased blood‐brain barrier permeability is associated with dementia and diabetes but not amyloid pathology or APOE genotype. Neurobiol Aging. 2017;51:104‐112. 28061383 10.1016/j.neurobiolaging.2016.11.017 PMC5754327

[119] Qiao J , Lawson CM , Rentrup KFG , Kulkarni P , Ferris CF . Evaluating blood‐brain barrier permeability in a rat model of type 2 diabetes. J Transl Med. 2020;18(1):256. 32580725 10.1186/s12967-020-02428-3 PMC7313122

[120] Huber JD , VanGilder RL , Houser KA . Streptozotocin‐induced diabetes progressively increases blood‐brain barrier permeability in specific brain regions in rats. Am J Physiol Heart Circ Physiol. 2006;291(6):H2660‐H2668. 16951046 10.1152/ajpheart.00489.2006

[121] Salameh TS , Mortell WG , Logsdon AF , Butterfield DA , Banks WA . Disruption of the hippocampal and hypothalamic blood‐brain barrier in a diet‐induced obese model of type II diabetes: prevention and treatment by the mitochondrial carbonic anhydrase inhibitor, topiramate. Fluids Barriers CNS. 2019;16(1):1. 30616618 10.1186/s12987-018-0121-6 PMC6323732

[122] Pivoriūnas A , Verkhratsky A . Astrocyte‐endotheliocyte axis in the regulation of the blood‐brain barrier. Neurochem Res. 2021;46(10):2538‐2550. 33961207 10.1007/s11064-021-03338-6

[123] Heithoff BP , George KK , Phares AN , Zuidhoek IA , Munoz‐Ballester C , Robel S . Astrocytes are necessary for blood‐brain barrier maintenance in the adult mouse brain. Glia. 2021;69(2):436‐472. 32955153 10.1002/glia.23908 PMC7736206

[124] Guérit S , Fidan E , Macas J , et al. Astrocyte‐derived Wnt growth factors are required for endothelial blood‐brain barrier maintenance. Prog Neurobiol. 2021;199:101937. 33383106 10.1016/j.pneurobio.2020.101937

[125] Haruwaka K , Ikegami A , Tachibana Y , et al. Dual microglia effects on blood brain barrier permeability induced by systemic inflammation. Nat Commun. 2019;10(1):5816. 31862977 10.1038/s41467-019-13812-z PMC6925219

[126] Ayala‐Guerrero L , García‐delaTorre P , Sánchez‐García S , Guzmán‐Ramos K . Serum levels of glial fibrillary acidic protein association with cognitive impairment and type 2 diabetes. Arch Med Res. 2022;53(5):501‐507. 35794041 10.1016/j.arcmed.2022.06.001

[127] Garvin J , Semenikhina M , Liu Q , et al. Astrocytic responses to high glucose impair barrier formation in cerebral microvessel endothelial cells. Am J Physiol Regul Integr Comp Physiol. 2022;322(6):R571‐R580. 35412389 10.1152/ajpregu.00315.2020 PMC9109795

[128] Bhattarai Y . Microbiota‐gut‐brain axis: interaction of gut microbes and their metabolites with host epithelial barriers. Neurogastroenterol Motil. 2018;30(6):e13366. 29878576 10.1111/nmo.13366

[129] Braniste V , Al‐Asmakh M , Kowal C , et al. The gut microbiota influences blood‐brain barrier permeability in mice. Sci Transl Med. 2014;6(263):263ra158. 10.1126/scitranslmed.3009759 PMC4396848 25411471

[130] Thaiss CA , Levy M , Grosheva I , et al. Hyperglycemia drives intestinal barrier dysfunction and risk for enteric infection. Science (New York, NY). 2018;359(6382):1376‐1383. 10.1126/science.aar3318 29519916

[131] Liu S , Gao J , Liu K , Zhang HL . Microbiota‐gut‐brain axis and Alzheimer's disease: implications of the blood‐brain barrier as an intervention target. Mech Ageing Dev. 2021;199:111560. 34411603 10.1016/j.mad.2021.111560

[132] Zhang J , Takahashi HK , Liu K , et al. Anti‐high mobility group box‐1 monoclonal antibody protects the blood‐brain barrier from ischemia‐induced disruption in rats. Stroke. 2011;42(5):1420‐1428. 21474801 10.1161/STROKEAHA.110.598334

[133] Varatharaj A , Galea I . The blood‐brain barrier in systemic inflammation. Brain Behav Immun. 2017;60:1‐12. 26995317 10.1016/j.bbi.2016.03.010

[134] Cunnane SC , Trushina E , Morland C , et al. Brain energy rescue: an emerging therapeutic concept for neurodegenerative disorders of ageing. Nat Rev Drug Discov. 2020;19(9):609‐633. 32709961 10.1038/s41573-020-0072-x PMC7948516

[135] Contreras CM , Gutiérrez‐García AG . Cognitive impairment in diabetes and poor glucose utilization in the intracellular neural milieu. Med Hypotheses. 2017;104:160‐165. 28673577 10.1016/j.mehy.2017.06.007

[136] Pardridge WM , Triguero D , Farrell CR . Downregulation of blood‐brain barrier glucose transporter in experimental diabetes. Diabetes. 1990;39(9):1040‐1044. 2384187 10.2337/diab.39.9.1040

[137] Wardzinski EK , Kistenmacher A , Melchert UH , Jauch‐Chara K , Oltmanns KM . Impaired brain energy gain upon a glucose load in obesity. Metab Clin Exp. 2018;85:90‐96. 29522772 10.1016/j.metabol.2018.02.013

[138] Song X , Zhu Z , Qian X , Liu X , Chen S , Tang H . Multi‐omics characterization of type 2 diabetes mellitus‐induced cognitive impairment in the db/db mouse model. Molecules (Basel, Switzerland). 2022;27(6):1904. 35335269 10.3390/molecules27061904 PMC8951264

[139] Huang S , Wang Y , Gan X , et al. Drp1‐mediated mitochondrial abnormalities link to synaptic injury in diabetes model. Diabetes. 2015;64(5):1728‐1742. 25412623 10.2337/db14-0758 PMC4407851

[140] Andersen JV , Christensen SK , Nissen JD , Waagepetersen HS . Improved cerebral energetics and ketone body metabolism in db/db mice. J Cereb Blood Flow Metab. 2017;37(3):1137‐1147. 28058963 10.1177/0271678X16684154 PMC5363491

[141] Girault FM , Sonnay S , Gruetter R , Duarte JMN . Alterations of brain energy metabolism in type 2 diabetic Goto‐Kakizaki rats measured In vivo by (13)C magnetic resonance spectroscopy. Neurotox Res. 2019;36(2):268‐278. 28971314 10.1007/s12640-017-9821-y

[142] Soto M , Herzog C , Pacheco JA , et al. Gut microbiota modulate neurobehavior through changes in brain insulin sensitivity and metabolism. Mol Psychiatry. 2018;23(12):2287‐2301. 29910467 10.1038/s41380-018-0086-5 PMC6294739

[143] Liu Z , Dai X , Zhang H , et al. Gut microbiota mediates intermittent‐fasting alleviation of diabetes‐induced cognitive impairment. Nat Commun. 2020;11(1):855. 32071312 10.1038/s41467-020-14676-4 PMC7029019

[144] Higarza SG , Arboleya S , Arias JL , Gueimonde M , Arias N . *Akkermansia muciniphila* and environmental enrichment reverse cognitive impairment associated with high‐fat high‐cholesterol consumption in rats. Gut Microbes. 2021;13(1):1‐20. 10.1080/19490976.2021.1880240 PMC7946069 33678110

[145] Margineanu MB , Sherwin E , Golubeva A , et al. Gut microbiota modulates expression of genes involved in the astrocyte‐neuron lactate shuttle in the hippocampus. Eur Neuropsychopharmacol. 2020;41:152‐159. 33191074 10.1016/j.euroneuro.2020.11.006

[146] Gao H , Jiang Q , Ji H , Ning J , Li C , Zheng H . Type 1 diabetes induces cognitive dysfunction in rats associated with alterations of the gut microbiome and metabolomes in serum and hippocampus. Biochim Biophys Acta Mol Basis Dis. 2019;1865(12):165541. 31472216 10.1016/j.bbadis.2019.165541

[147] Draznin B , Aroda VR , Bakris G , et al. 16. Diabetes care in the hospital: standards of medical care in diabetes‐2022. Diabetes Care. 2022;45(Suppl 1):S244‐S253. 34964884 10.2337/dc22-S016

[148] Rizzo MR , Di Meo I , Polito R , et al. Cognitive impairment and type 2 diabetes mellitus: focus of SGLT2 inhibitors treatment. Pharmacol Res. 2022;176:106062. 35017046 10.1016/j.phrs.2022.106062

[149] Sun C , Xiao Y , Li J , et al. Nonenzymatic function of DPP4 in diabetes‐associated mitochondrial dysfunction and cognitive impairment. Alzheimer's Dement. 2022;18(5):966‐987. 34374497 10.1002/alz.12437

[150] Samaras K , Makkar S , Crawford JD , et al. Metformin use is associated with slowed cognitive decline and reduced incident dementia in older adults with type 2 diabetes: the Sydney memory and ageing study. Diabetes Care. 2020;43(11):2691‐2701. 32967921 10.2337/dc20-0892

[151] Cukierman‐Yaffe T , Gerstein HC , Colhoun HM , et al. Effect of dulaglutide on cognitive impairment in type 2 diabetes: an exploratory analysis of the REWIND trial. Lancet Neurol. 2020;19(7):582‐590. 32562683 10.1016/S1474-4422(20)30173-3

[152] Moore EM , Mander AG , Ames D , et al. Increased risk of cognitive impairment in patients with diabetes is associated with metformin. Diabetes Care. 2013;36(10):2981‐2987. 24009301 10.2337/dc13-0229 PMC3781568

[153] Mao YF , Guo Z , Zheng T , et al. Intranasal insulin alleviates cognitive deficits and amyloid pathology in young adult APPswe/PS1dE9 mice. Aging Cell. 2016;15(5):893‐902. 27457264 10.1111/acel.12498 PMC5013027

[154] Park JS , Kam TI , Lee S , et al. Blocking microglial activation of reactive astrocytes is neuroprotective in models of Alzheimer's disease. Acta Neuropathol Commun. 2021;9(1):78. 33902708 10.1186/s40478-021-01180-z PMC8074239

[155] Shan Y , Tan S , Lin Y , et al. The glucagon‐like peptide‐1 receptor agonist reduces inflammation and blood‐brain barrier breakdown in an astrocyte‐dependent manner in experimental stroke. J Neuroinflammation. 2019;16(1):242. 31779652 10.1186/s12974-019-1638-6 PMC6883580

[156] Lam YY , Tsai SF , Chen PC , Kuo YM , Chen YW . Pioglitazone rescues high‐fat diet‐induced depression‐like phenotypes and hippocampal astrocytic deficits in mice. Biomed Pharmacother. 2021;140:111734. 34022606 10.1016/j.biopha.2021.111734

[157] Kushwaha R , Mishra J , Gupta AP , et al. Rosiglitazone up‐regulates glial fibrillary acidic protein via HB‐EGF secreted from astrocytes and neurons through PPARγ pathway and reduces apoptosis in high‐fat diet‐fed mice. J Neurochem. 2019;149(5):679‐698. 30311190 10.1111/jnc.14610

[158] Xiao S , Chan P , Wang T , et al. A 36‐week multicenter, randomized, double‐blind, placebo‐controlled, parallel‐group, phase 3 clinical trial of sodium oligomannate for mild‐to‐moderate Alzheimer's dementia. Alzheimer's Res Ther. 2021;13(1):62. 33731209 10.1186/s13195-021-00795-7 PMC7967962

[159] Giugliano D , Maiorino MI , Esposito K . Intensive lifestyle intervention for type 2 diabetes. Jama. 2017;318(24):2494. 10.1001/jama.2017.17232 29279921

[160] Olson CA , Iñiguez AJ , Yang GE , et al. Alterations in the gut microbiota contribute to cognitive impairment induced by the ketogenic diet and hypoxia. Cell Host Microbe. 2021;29(9):1378‐1392.e1376. 34358434 10.1016/j.chom.2021.07.004 PMC8429275

[161] Nagpal R , Neth BJ , Wang S , Mishra SP , Craft S , Yadav H . Gut mycobiome and its interaction with diet, gut bacteria and Alzheimer's disease markers in subjects with mild cognitive impairment: a pilot study. EBioMedicine. 2020;59:102950. 32861197 10.1016/j.ebiom.2020.102950 PMC7475073

[162] Lutomska LM , Miok V , Krahmer N , et al. Diet triggers specific responses of hypothalamic astrocytes in time and region dependent manner. Glia. 2022;70(11):2062‐2078. 35802021 10.1002/glia.24237

[163] Düking T , Spieth L , Berghoff SA , et al. Ketogenic diet uncovers differential metabolic plasticity of brain cells. Sci Adv. 2022;8(37):eabo7639. 36112685 10.1126/sciadv.abo7639 PMC9481126

[164] Olson CA , Vuong HE , Yano JM , Liang QY , Nusbaum DJ , Hsiao EY . The gut microbiota mediates the anti‐seizure effects of the ketogenic diet. Cell. 2018;173(7):1728‐1741.e1713. 29804833 10.1016/j.cell.2018.04.027 PMC6003870

[165] Gregosa A , Vinuesa Á , Todero MF , et al. Periodic dietary restriction ameliorates amyloid pathology and cognitive impairment in PDAPP‐J20 mice: potential implication of glial autophagy. Neurobiol Dis. 2019;132:104542. 31351172 10.1016/j.nbd.2019.104542

[166] Vallee KJ , Fields JA . Caloric restriction mimetic 2‐deoxyglucose reduces inflammatory signaling in human astrocytes: implications for therapeutic Strategies targeting neurodegenerative diseases. Brain Sci. 2022;12(3):308. 35326266 10.3390/brainsci12030308 PMC8945872

[167] Wang Y , Dilidaxi D , Wu Y , Sailike J , Sun X , Nabi XH . Composite probiotics alleviate type 2 diabetes by regulating intestinal microbiota and inducing GLP‐1 secretion in db/db mice. Biomed Pharmacother. 2020;125:109914. 32035395 10.1016/j.biopha.2020.109914

[168] Zhang Z , Liang X , Lv Y , et al. Evaluation of probiotics for improving and regulation metabolism relevant to type 2 diabetes in vitro. J Funct Foods. 2020;64:103664.

[169] Davari S , Talaei SA , Alaei H , Salami M . Probiotics treatment improves diabetes‐induced impairment of synaptic activity and cognitive function: behavioral and electrophysiological proofs for microbiome‐gut‐brain axis. Neuroscience. 2013;240:287‐296. 23500100 10.1016/j.neuroscience.2013.02.055

[170] Morshedi M , Saghafi‐Asl M , Hosseinifard ES . The potential therapeutic effects of the gut microbiome manipulation by synbiotic containing‐*Lactobacillus plantarum* on neuropsychological performance of diabetic rats. J Transl Med. 2020;18(1):18. 31924200 10.1186/s12967-019-02169-y PMC6953298

[171] Ji L , Tong X , Wang H , et al. Efficacy and safety of traditional chinese medicine for diabetes: a double‐blind, randomised, controlled trial. PLoS One. 2013;8(2):e56703. 23460810 10.1371/journal.pone.0056703 PMC3584095

[172] Vellios N , van der Zee K . Dataset on cigarette smokers in six south African townships. Data Brief. 2020;32:106260. 32964081 10.1016/j.dib.2020.106260 PMC7490728

[173] Yue SJ , Wang WX , Yu JG , et al. Gut microbiota modulation with traditional Chinese medicine: a system biology‐driven approach. Pharmacol Res. 2019;148:104453. 31541688 10.1016/j.phrs.2019.104453

[174] Meng J , Zhu Y , Ma H , Wang X , Zhao Q . The role of traditional Chinese medicine in the treatment of cognitive dysfunction in type 2 diabetes. J Ethnopharmacol. 2021;280:114464. 34329715 10.1016/j.jep.2021.114464

[175] Shao J , Ma X , Qu L , Ma P , Huang R , Fan D . Ginsenoside Rh4 remodels the periphery microenvironment by targeting the brain‐gut axis to alleviate depression‐like behaviors. Food Chem. 2022;404(Pt B):134639. 36283312 10.1016/j.foodchem.2022.134639

[176] Liu Y , Liu W , Li J , et al. A polysaccharide extracted from *Astragalus membranaceus* residue improves cognitive dysfunction by altering gut microbiota in diabetic mice. Carbohydr Polym. 2019;205:500‐512. 30446134 10.1016/j.carbpol.2018.10.041

[177] Dun C , Liu J , Qiu F , et al. Effects of Astragalus polysaccharides on memory impairment in a diabetic rat model. Neuropsychiatr Dis Treat. 2016;12:1617‐1621. 27445477 10.2147/NDT.S106123 PMC4936836

[178] Chen X , Chen C , Fu X . Hypoglycemic effect of the polysaccharides from *Astragalus membranaceus* on type 2 diabetic mice based on the "gut microbiota‐mucosal barrier". Food Funct. 2022;13(19):10121‐10133. 36106494 10.1039/d2fo02300h

[179] Huang YC , Tsay HJ , Lu MK , et al. Astragalus membranaceus‐polysaccharides ameliorates obesity, hepatic steatosis, neuroinflammation and cognition impairment without affecting amyloid deposition in metabolically stressed APPswe/PS1dE9 mice. Int J Mol Sci. 2017;18(12):2746. 29258283 10.3390/ijms18122746 PMC5751345

[180] Cheng H , Liu J , Tan Y , Feng W , Peng C . Interactions between gut microbiota and berberine, a necessary procedure to understand the mechanisms of berberine. J Pharm Anal. 2022;12(4):541‐555. 36105164 10.1016/j.jpha.2021.10.003 PMC9463479

[181] Moghaddam HK , Baluchnejadmojarad T , Roghani M , et al. Berberine ameliorate oxidative stress and astrogliosis in the hippocampus of STZ‐induced diabetic rats. Mol Neurobiol. 2014;49(2):820‐826. 24113841 10.1007/s12035-013-8559-7

[182] Liu M , Gao L , Zhang N . Berberine reduces neuroglia activation and inflammation in streptozotocin‐induced diabetic mice. Int J Immunopathol Pharmacol. 2019;33:2058738419866379. 31337260 10.1177/2058738419866379 PMC6657114

[183] Miao C , Chen H , Li Y , et al. Curcumin and its analog alleviate diabetes‐induced damages by regulating inflammation and oxidative stress in brain of diabetic rats. Diabetol Metab Syndr. 2021;13(1):21. 33602334 10.1186/s13098-021-00638-3 PMC7891034

[184] Faheem NM , El Askary A . Neuroprotective role of curcumin on the hippocampus against the structural and serological alterations of streptozotocin‐induced diabetes in Sprague Dawely rats. Iran J Basic Med Sci. 2017;20(6):690‐699. 28868124 10.22038/IJBMS.2017.8839 PMC5569451

[185] Huang J , Guan B , Lin L , Wang Y . Improvement of intestinal barrier function, gut microbiota, and metabolic endotoxemia in type 2 diabetes rats by curcumin. Bioengineered. 2021;12(2):11947‐11958. 34818970 10.1080/21655979.2021.2009322 PMC8810160

[186] Lin CH , Chou CC , Lee YH , Hung CC . Curcumin facilitates aryl hydrocarbon receptor activation to ameliorate inflammatory astrogliosis. Molecules (Basel, Switzerland). 2022;27(8):2507. 35458704 10.3390/molecules27082507 PMC9024799

